# Stability of the human gut virome and effect of gluten-free diet

**DOI:** 10.1016/j.celrep.2021.109132

**Published:** 2021-05-18

**Authors:** Sanzhima Garmaeva, Anastasia Gulyaeva, Trishla Sinha, Andrey N. Shkoporov, Adam G. Clooney, Stephen R. Stockdale, Johanne E. Spreckels, Thomas D.S. Sutton, Lorraine A. Draper, Bas E. Dutilh, Cisca Wijmenga, Alexander Kurilshikov, Jingyuan Fu, Colin Hill, Alexandra Zhernakova

**Affiliations:** 1 Department of Genetics, University of Groningen, University Medical Center Groningen, Groningen 9713GZ, the Netherlands; 2 APC Microbiome Ireland and School of Microbiology, University College Cork, Cork T12 YT20, Ireland; 3 Department of Pediatrics, University of Groningen, University Medical Center Groningen, Groningen 9713GZ, the Netherlands; 4 Theoretical Biology and Bioinformatics, Science for Life, Utrecht University, Utrecht 3584 CH, the Netherlands

## Abstract

The human gut microbiome consists of bacteria, archaea, eukaryotes, and viruses. The gut viruses are relatively underexplored. Here, we longitudinally analyzed the gut virome composition in 11 healthy adults: its stability, variation, and the effect of a gluten-free diet. Using viral enrichment and a *de novo* assembly-based approach, we demonstrate the quantitative dynamics of the gut virome, including dsDNA, ssDNA, dsRNA, and ssRNA viruses. We observe highly divergent individual viral communities, carrying on an average 2,143 viral genomes, 13.1% of which were present at all 3 time points. In contrast to previous reports, the *Siphoviridae* family dominates over *Microviridae* in studied individual viromes. We also show individual viromes to be stable at the family level but to vary substantially at the genera and species levels. Finally, we demonstrate that lower initial diversity of the human gut virome leads to a more pronounced effect of the dietary intervention on its composition.

## Graphical abstract

**Graphical abstract:**
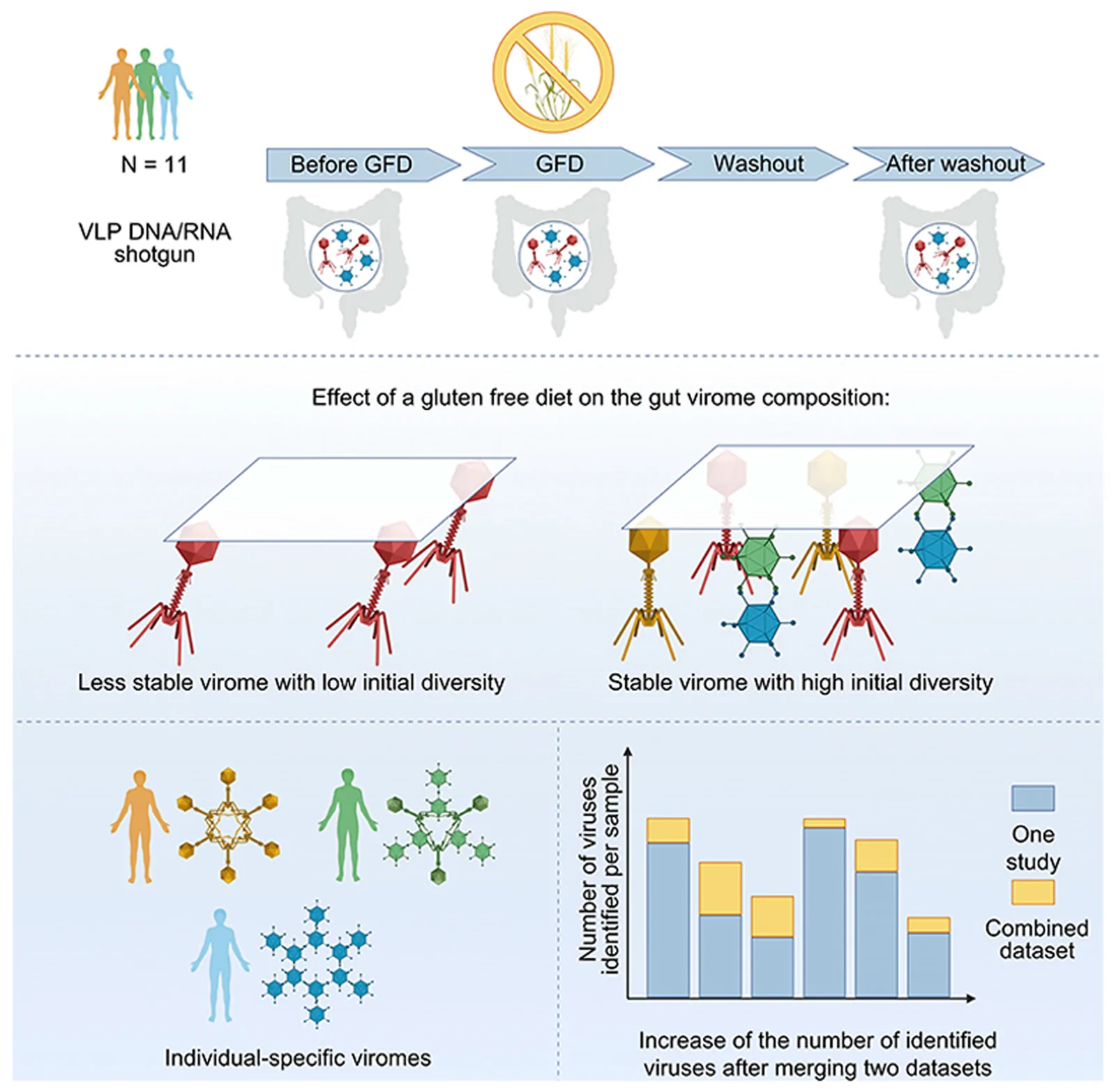

## Introduction

The human gut microbiome has been linked to many diseases and conditions and is influenced by various host and environmental factors (Falony et al., 2016; Rothschild et al., 2018; Zhernakova et al., 2016). However, our understanding of the role of the gut virome in human health is far less extensive, even though virome is an essential component of the gut ecosystem. The estimated ratio of virus-like particles (VLPs) to bacteria in the gut is ~1:1, and many viruses occur as integrated prophages in the genomes of bacteria (Hoyles et al., 2014; Sender et al., 2016; Shkoporov and Hill, 2019; Shkoporov et al., 2018, 2019).

Wide-scale studies of the gut virome are limited by multiple technical and methodological challenges (Garmaeva et al., 2019). First, the protocols for extracting genetic material from VLPs from stool samples are laborious and require more time than isolation of total DNA. Second, the lack of a universal viral marker gene comparable to the 16S rRNA gene in bacteria significantly complicates taxonomy-focused ecological studies. Third, currently available viral reference databases are incomplete, and a substantial fraction of the sequences in viromic datasets remains uncharacterized and constitute so-called viral dark matter (Roux et al., 2015a). As a result, virome studies have thus far been performed on a relatively small scale. Despite these challenges, several studies have indicated the association of the gut virome with various diseases, including inflammatory bowel disease (Clooney et al., 2019; Norman et al., 2015), colorectal cancer (Nakatsu et al., 2018), type 1 and type 2 diabetes (Ma et al., 2018; Zhao et al., 2017), malnutrition (Reyes et al., 2015), acquired immune deficiency syndrome (Monaco et al., 2016), and Parkinson’s disease (Tetz et al., 2018). In addition, successful treatment of *Clostridium difficile*-infected patients using fecal filtrate rather than full fecal microbiota transplant hints at a possible role for the virome and other filtrate components, such as the metabolome, in microbiome recovery after infection (Ott et al., 2017).

A recent longitudinal study in 10 healthy Irish volunteers over the course of 1 year revealed the temporal stability and individual specificity of the human gut virome (Shkoporov et al., 2019) in the absence of any intervention, which is in line with earlier studies (Minot et al., 2013; Reyes et al., 2010). This Irish study found that a major proportion of the virome was individual specific and remained stable across 12 months, the persistent personal virome (PPV), while a smaller proportion was less stable and shared between more individuals (transiently detected virome [TDV]). The study also demonstrated the high variation of the virome across individuals, which likely reflects the effects of multiple environmental and intrinsic factors, as has been previously shown for the virome (Reyes et al., 2010) and for the gut bacterial communities.

As the stability of the gut virome under the influence of external factors is relatively underexplored, we aimed to study the effect of a gluten-free diet (GFD) on virome composition. Gluten is the storage protein of wheat, barley, and rye. Exclusion of gluten-containing products from the diet is the only treatment for celiac disease, a common food sensitivity that affects ~1% of the population worldwide (Sollid, 2002). However, a GFD is also becoming one of the most popular diets (Newberry et al., 2017) and is being followed by individuals with various gut complaints and by healthy individuals aiming to lose weight or improve health (Pearlman and Casey, 2019; Vazquez-Roque et al., 2013). Several studies have indicated that a gluten-free or low-gluten diet changes the gut bacterial composition (Bonder et al., 2016; Hansen et al., 2018; De Palma et al., 2009).

**Figure 1:**
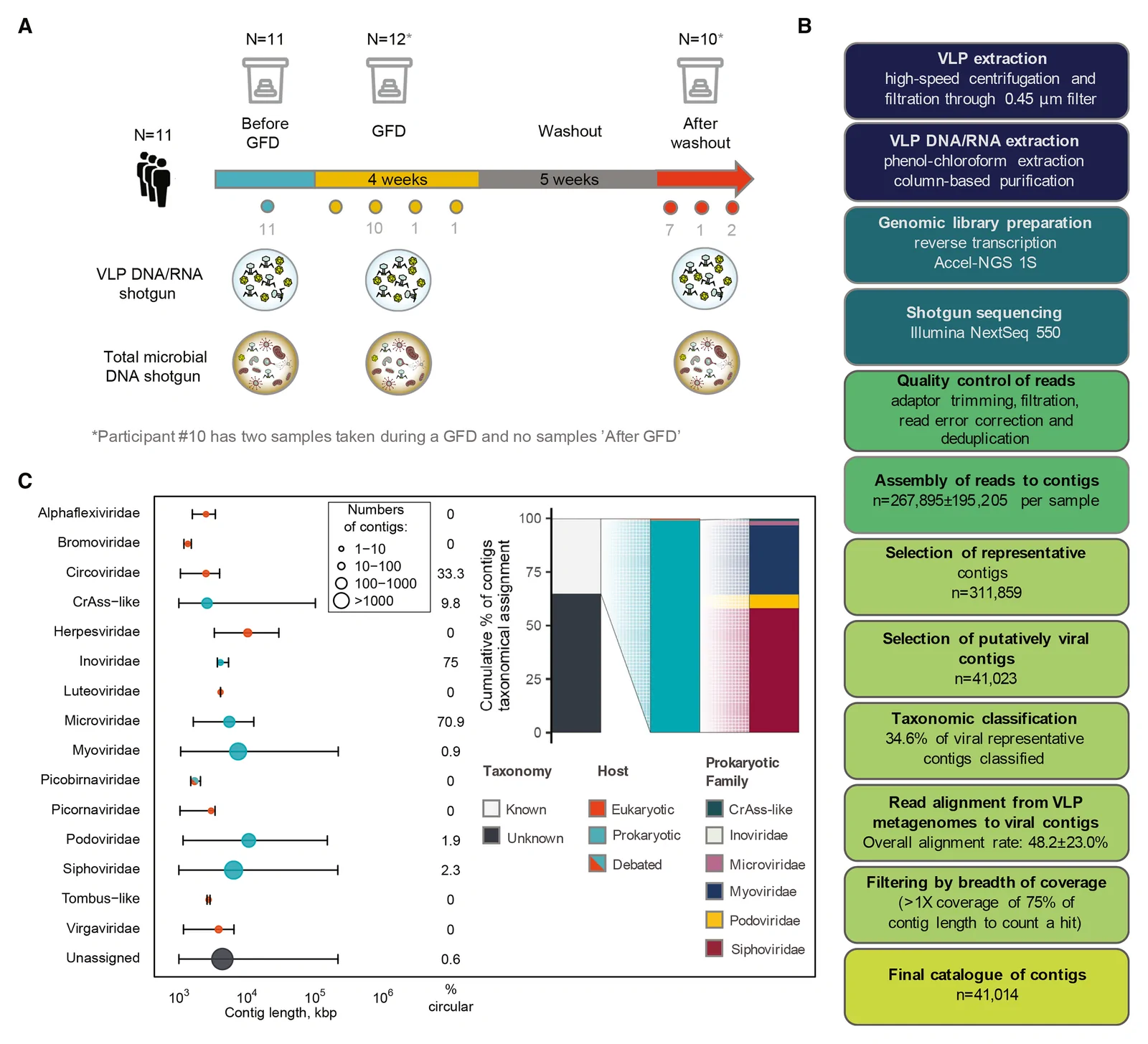
Experimental design and distribution of viral representative contigs by length, taxonomic family, and host (A)Timeline of fecal sample collection from 11 study subjects and types of analyses performed. The number of samples collected per the time point is indicated with colored dots. One participant (no. 10) was sampled twice during the GFD, with no sample taken after the washout period. (B) Overview of the experimental protocols and bioinformatic pipelines. See Figure S1 for the detailed bioinformatic pipeline. (C) Distribution of 41,014 viral-representative contigs by length, taxonomic family, and host. Segments represent the spread between the minimal and maximal lengths of contigs assigned to the taxonomic family rank. Dot size and color represent the number of contigs within the taxonomic family rank and the host of the viruses, respectively. Numbers opposite each segment represent the percentage of circular contigs among the contigs within the taxonomic family rank. Bar plots show the cumulative percentage of contigs that have taxonomic and host assignments. Notably, these numbers represent only the diversity based on the number of contigs. Note that the host of picobirnaviruses is debated (Krishnamurthy and Wang, 2018).

We analyzed the gut virome in 11 individuals at 3 time points: before, during, and 5 weeks after GFD intervention (Figure 1A). More specifically, we investigated the composition and stability of the gut virome across the three time points, compared the virome and microbiome compositions, and explored the effect of the GFD on the virome composition. Importantly, we sequenced VLP metagenomes without amplification, which allowed us to avoid amplification bias and accurately estimate the virome composition. We thereby redefined the composition of the PPV, observed trends toward changes in the human gut virome during the dietary intervention, and confirmed the overall resilience of a more diverse gut ecosystem. In addition, we demonstrate that combining the viral contigs reconstructed in our study with those from Shkoporov et al. (2019) allowed us to identify more viruses within the VLP metagenomes.

## Results

### Study design

To determine the stability of the gut virome in response to dietary changes, we monitored the fecal viromes of 11 healthy adults who followed a GFD (Bonder et al., 2016). Fecal samples were collected at 3 time points: before the GFD, during the GFD, and after a 5-week washout period (Figure 1A). Genomic DNA from the total microbial community and DNA and RNA from VLPs were isolated from samples and sequenced without amplification, making this one of the largest quantitative virome studies of the human gut to date (Kang et al., 2017). Virome composition of the VLP metagenomes was established using a *de novo* assembly-based approach (Figures 1B and S1) described by Shkoporov et al. (2019). The potential contamination of VLP metagenomes with reads of bacterial origin was estimated to be low (median 6.0% per sample; Figure S2A) based on the fraction of reads (median of 1.9 × 10^−5^% per sample) aligning to the conserved single-copy bacterial *cpn60* chaperonin (Shkoporov et al., 2018, 2019). Detailed descriptions of the total community and VLP metagenome isolation, sequencing, and analysis are provided in the method details section.

### Variability in size and topology of genomes in the human gut virome

We identified 41,014 viral contigs using the *de novo* assembly-based approach (Shkoporov et al., 2019), with the addition of a RNA-dependent RNA polymerase (RdRp) domain search (see method details). The viral contigs made up 13.2% of the total set of dereplicated contigs longer than 1 kb. These viral-representative contigs formed the custom viral database of this study and were used in all of the subsequent analyses. Approximately 96% of viral-representative contigs were 1–25 kbp in length, 4% were 25–200 kbp, and fewer than 0.01% were longer than 200 kbp (Figure S2B; Table S1). No complete genomes of the recently identified huge phages (>200 kbp) from Al-Shayeb et al. (2020) were re-constructed in our VLP metagenomes, although parts of huge phage genomes were detected at 50% identity over 90% of the length of the representative contig. Only 1.2% (n = 509) of the 41,014 viral-representative contigs had identical ends, which suggests that they represented complete genomes of viruses with circular or terminally redundant linear genomes (Figure 1C). The circular contigs varied in size from 3 to >200 kbp, with 75% of circular contigs being 3–41.1 kbp in length (Figure S2C), which is consistent with previous studies (Al-Shayeb et al., 2020).

### Taxonomic composition of the human gut viromes

Even in a well-studied environment like the human microbiome, the vast majority of viruses have not yet been taxonomically classified and approved by the International Committee on Taxonomy of Viruses (ICTV). Thus, the taxonomic interpretation of viromic datasets remains challenging. Of the 41,014 identified viral genomes and fragments, only 225 had close homologs (>50% nucleotide identity over 90% of sequence length) among previously described viruses in the Viral RefSeq database (release no. 98). These mainly included representative contigs with homology to *Lactococcus* (30 different strains) and *Leuconostoc* phages, crAss-like phages, some eukaryotic single-stranded DNA (ssDNA) viruses, and plant viruses. This supports earlier evidence that only a tiny percentage of viral genomes have annotated reference genomes (Aggarwala et al., 2017).

To gain a more complete view of the composition of the viromes, we used a combination of Demovir assignments (https://github.com/feargalr/Demovir) and vConTACT2 clustering pipelines (Bin Jang et al., 2019), as described in Method details. This approsach allowed taxonomic assignment to four orders approved by ICTV for 34.6% of our 41,014 contigs, as well as assignment to 15 prokaryotic and eukaryotic families of double-stranded DNA (dsDNA), ssDNA, dsRNA, and ssRNA viruses (Table S2).

The majority of taxonomically classified viral-representative contigs were assigned to families of bacteriophages (dsDNA and ssDNA prokaryotic viruses; 99.2%), while the remaining 0.8% of viral contigs were split among dsDNA and ssDNA (0.4%) and dsRNA and ssRNA (0.4%) eukaryotic viruses (Figure 1C), which is in line with previous findings (Kim et al., 2011; Minot et al., 2013; Reyes et al., 2010; Waller et al., 2014). The majority of the viral-representative contigs with assigned taxonomy belonged to the bacteriophage order *Caudovirales* (98.1%). On the family level, the prokaryotic viruses mainly binned to the families *Siphoviridae, Myoviridae, Podoviridae, Microviridae*, crAss-like phages, and *Inoviridae* (Figure 1C). Eukaryotic viruses were more diverse at the family level and included potential viruses of humans (*Circoviridae* and *Herpesviridae*) and plants (*Alphaflexiviridae, Bromoviridae, Luteoviridae*, and *Virgaviridae*) (Figure 1C). Up to 75% of viral-representative contigs assigned to families such as *Microviridae, Inoviridae*, and *Circoviridae*, known to have small circular genomes, were circularized and thus suggestively complete (Figure 1C).

**Figure 2:**
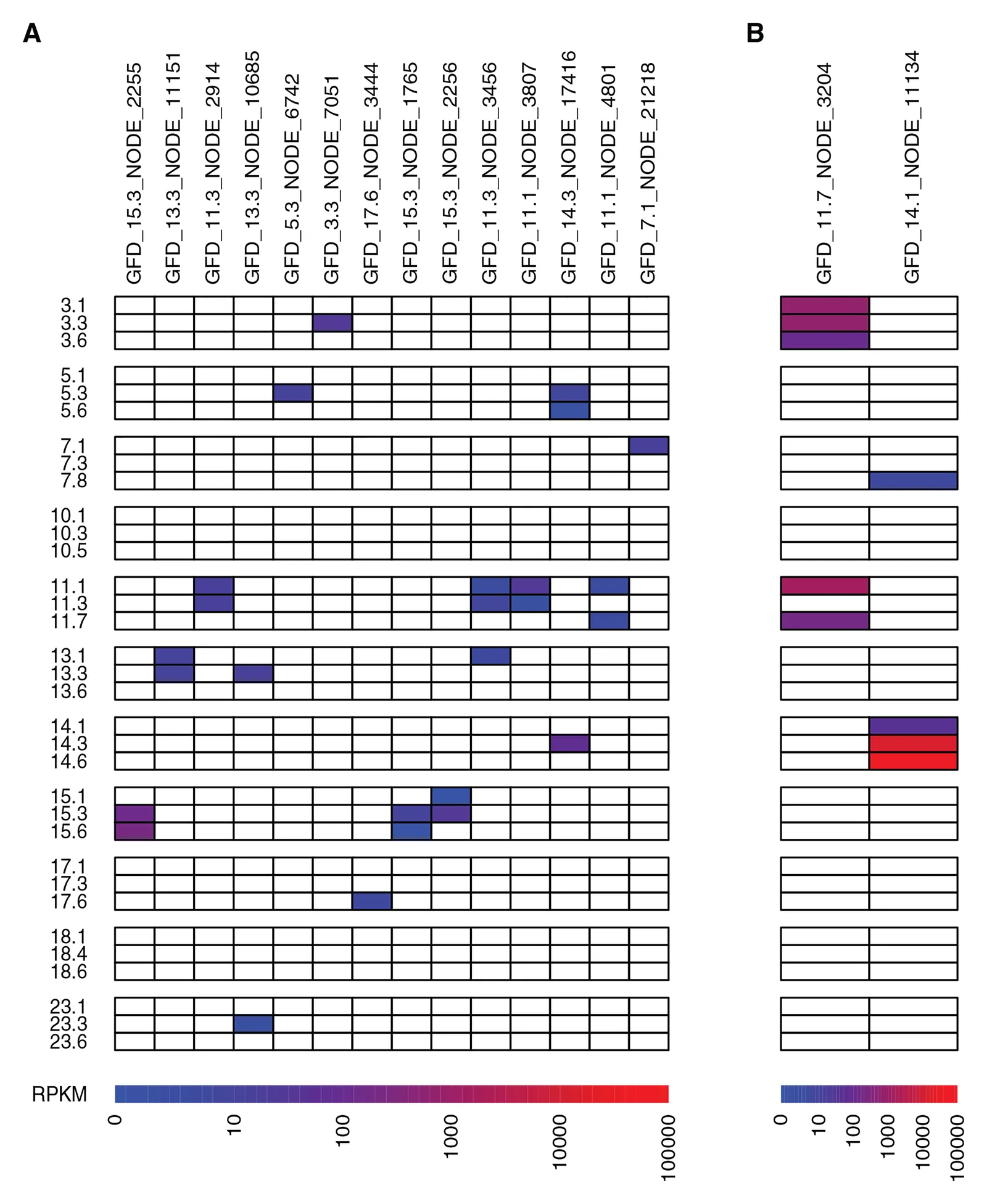
Abundance of two groups of RNA viruses (A) Abundance of picobirnavirus contigs in samples. (B) Abundance of tombus-like contigs in samples. Non-zero RPKM read count values are indicated by color. See also Table S3.

The VLP metagenome extraction and sequencing protocol used in this study offered a rare opportunity to analyze the RNA viruses of the gut. In our dataset, RNA viruses made up 0.4% of all taxonomically assigned viral-representative contigs. Using the presence of RdRp as a marker for contigs representing RNA viruses (Ahlquist, 2002; Shi et al., 2016; Wolf et al., 2018), we detected both known RNA viruses (picornavirus *Aichi virus A* in one sample and diverse plant viruses in multiple samples; Table S3) and divergent RNA viruses that may represent new species (14 picobirnaviruses and 2 putative tombus-like viruses, Figures S3 and S4; Table S3). The identified picobirnavirus contigs represent segment 2 of picobirnavirus genomes (Figure S3) and fall within the genogroups 1 and 2 of the family *Picobirnaviridae* (Figure S5). Picobirnavirus contigs were identified in 15 samples, nearly half of them (7) taken during the GFD (Figure 2A), suggesting a possible influence of the GFD on the picobirnavirus fraction of the gut virome. The two tombus-like contigs encoded RdRp in a central open reading frame (ORF) flanked by smaller ORFs (Figure S4). These representative contigs received taxonomic assignment based on the strong sequence similarity of their RdRp (e-value < 10^−50^; see Table S3) to that of the tombus-like viruses identified in a metatranscriptomics study of invertebrate hosts (Shi et al., 2016). Both were present in the samples from a few individuals, sometimes in extremely high quantities (maximum: 6.1 × 10^4^ reads per kilo-base per million reads [RPKM]; see Figure 2B).

In summary, taxonomy was assigned at the order rank for 34.6% of identified viruses and viral fragments in the curated viral database and at the family rank for 26%, 99.2% of the viral-representative contigs with known taxonomy represented bacteriophages, and 0.8% represented eukaryotic viruses, including RNA viruses.

### The structure of the human gut virome

**Figure 3:**
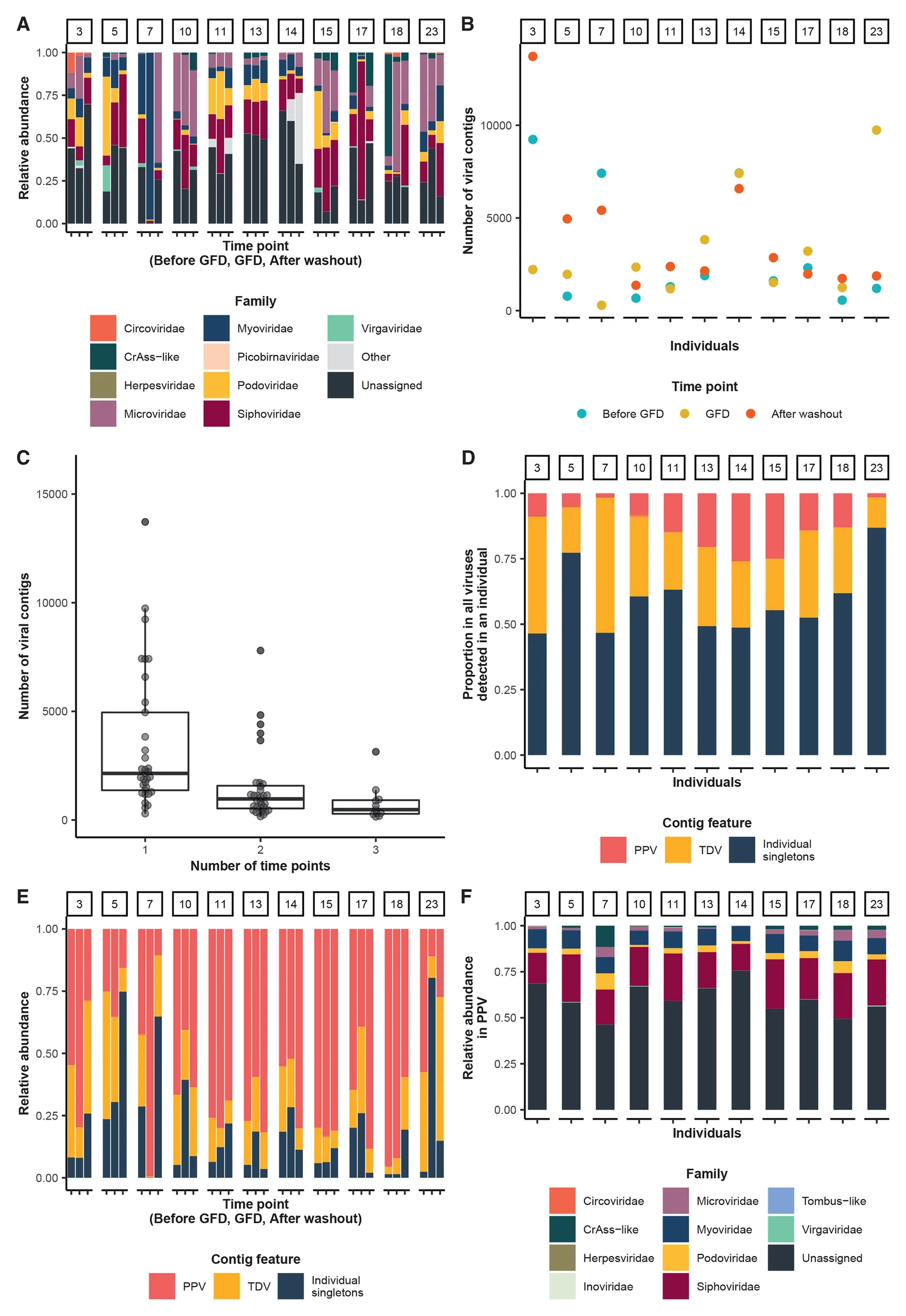
Individual virome community structure (A) Family-level taxonomic composition of viromes in 11 individuals by time point. Only viral families present in >10 samples are shown; the rest are pooled to the “Others” category. The RPKM counts are normalized to relative abundances (from 0 to 1). See also Figure S6 and Table S4. (B) Number of viral-representative contigs detected per time point per subject. Each dot represents 1 sample. Dot color indicates time point. (C) Number of viral-representative contigs per subject as a function of conservation (presence in a given number of time points). The first boxplot is based on the number of viral representative contigs in all samples. The second boxplot is based on pairs of time points (“before GFD” and “GFD,” “before GFD” and “after washout,” and “GFD” and “after washout”). The third boxplot is based on all 3 time points. All boxplots are standard Tukey type; see STAR Methods for details. (D) Fractions of personal persistent virome (PPV), transiently detected virome (TDV), and individual singletons for all of the viruses detected in an individual throughout the study. Numbers of viruses are normalized (from 0 to 1). (E) Cumulative relative abundance of viruses defined as PPV, TDV, and individual singletons in 11 individuals by time point. Pooled RPKM counts are normalized to relative abundances (from 0 to 1). (F) Taxonomic composition of viruses defined as PPV at the family level in 11 individuals. The RPKM counts are normalized to relative abundances (from 0 to 1).

We further aimed to analyze the individual fecal viral communities, which had, on average, 48.2% of reads mapped to the curated viral database per sample. On average, 9 viral families were detected per individual (Figures 3A and S6; Table S4), with members of the order *Caudovirales* dominating the fecal viral communities, with a median RPKM count value of 3.0 × 10^4^ ± 2.1 ×10^4^ per sample. The families *Myoviridae, Podoviridae*, and *Siphoviridae* from the order *Caudovirales* and family *Microviridae* were detected in every individual (Figure 3A). Among these, the family *Siphoviridae* was the most abundant, with a median RPKM count of 1.2 × 10^4^ (Figure 3A). The second most abundant family was *Microviridae*, with a median RPKM count of 5.5 × 10^3^ (minimum RPKM count of 1.1 × 10^2^, maximum of 9.1 × 10^4^). The crAss-like family was detected in 29 of 33 samples, with the crAss-like phages ERR844003_ms_1 (96 kbp, Guerin et al., 2018) and HvCF_D5_ms_5 (92 kbp) being the most prevalent. Among families of eukaryotic viruses, *Virgaviridae* and *Herpesviridae* were the most prevalent.

At the level of viral-representative contigs, 25.7% of all contigs detected in the dataset were found only once in one individual (read coverage of R75% of contig length was used to count a hit), whereas no contigs were shared across all individuals and all time points (Figure S7A). Only 10 viruses were present in more than 27 samples (80% of the samples) (Figure S7A), and 6 of these shared viruses were from the order *Caudovirales*. The median number of viruses identified per sample varied widely: from 292 to 13,717 viral genomes or genome fragments per sample (Figure 3B).

On average, 2,143 viral genomes or fragments were detected in each sample (Figure 3B). Meanwhile, the median number of all of the viruses detected per individual (in all 3 samples) was 4,636. Viral-representative contigs that were unique to a time point for every individual (i.e., individual singletons) composed more than half of all viruses detected in an individual (Figures 3C and 3D). The majority of individual singletons were not assigned to any viral family (median 70% per individual). In contrast, on average, only 13.1% of the viruses detected in an individual (median absolute number: 477) were shared across all 3 time points (Figures 3C and 3D) to form a PPV. Despite representing a small fraction of the overall viral diversity in each sample, viruses of the PPV recruited an average of 63.6% of sequencing reads per sample (Figure 3E), and this proportion did not change throughout the duration of the study. For the viruses in the PPV, taxonomy could be assigned for 40% of individual viruses, on average, with the most prevalent PPV viruses belonging to the families *Siphoviridae, Myoviridae*, and *Podoviridae* (median number of contigs assigned 22%, 9.2%, and 3.6% per individual, respectively) (Figure 3F). The rest of the viruses detected in an individual were composed of viruses shared across two time points (i.e., the TDV).

In summary, we observed high individual specificity of fecal viral communities, which is in line with previous reports (Moreno-Gallego et al., 2019; Reyes et al., 2010; Shkoporov et al., 2019). Despite this, as we went up in taxonomic rank, we saw more considerable overlap and less inter-individual variation in the different individual’s virome compositions. Specifically, 2.6% of contigs, 4.6% of genus-level virus clusters (VCs), and 62.5% of assigned virus families were shared among more than half of all individuals.

### Human gut virome is moderately altered by GFD

**Figure 4:**
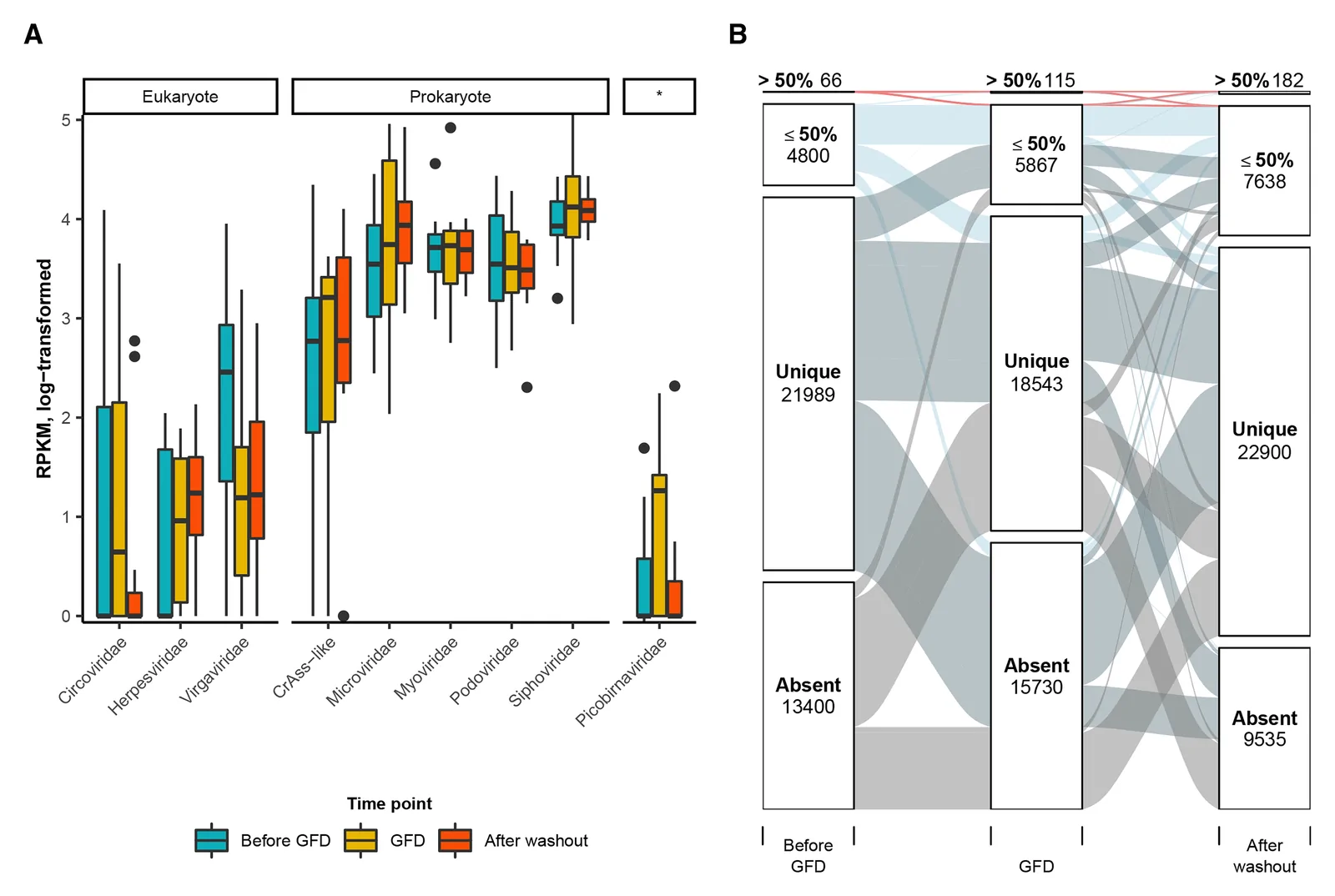
Stability of the human gut virome during a GFD at the family rank and the level of representative contigs (A) Dynamics of the most prevalent viral families throughout the study. Only families detected in at least 15 individuals are shown. The viral families are split based on the putative host. Note that the host of picobirnaviruses is debated. All boxplots are standard Tukey type; see STAR Methods for details. (B) Sankey diagram illustrating how the prevalence of viral-representative contigs changed upon transition from the first to second to third time point. Category (present in >50% of samples, in ≤50% of samples, unique, absent) and number of contigs are indicated in bold and plain fonts, respectively. Individual no. 10, who was not sampled after the washout, is excluded.

We next investigated the effect of a GFD on the virome composition. No concordant trend was observed for changes in alpha diversity at the viral family level during the dietary intervention. On RPKM counts, a few families showed trends toward changes in their relative abundances (Figure 4A). Nominal significance was detected in changes of the RPKM counts of the *Podoviridae* and crAss-like bacteriophage families, which showed a 2- and 4-fold decrease and increase in RPKM counts on a GFD, respectively (nominal p < 0.05; Figure 4A). Concordantly, we observed an increase in the abundance of the crAssphage host genus *Bacteroides* on the GFD (nominal p = 0.05; Figure S7B). The relative abundance of *Virgaviridae*, a family mainly composed of viruses that infect plants, including rye and wheat, decreased on the GFD (nominal p = 0.03; Figure 4A), with incomplete recovery after the washout period. Overall, these findings suggest that the gut virome remains stable at the family level during a GFD, with some fluctuations, although these trends require confirmation using larger datasets.

To explore the links between the changes in bacterial and viral communities during the study, we tested the covariation between the viral and microbial communities. To do so, we compared Bray-Curtis distance matrices for the two communities at the level of viral-representative contigs and bacterial species. The variation was positively correlated between the bacterial and viral communities (Mantel test, R = 0.36, p = 10^−4^; Figure S7C), which could be explained by the predominance of bacteriophages that infect bacteria in the human gut.

As most of the viral contigs were not taxonomically classified, we further investigated compositional changes at the viral-representative contig level. Here, we traced how the prevalence of each viral-representative contig changed among individuals at the time points “before GFD” to “GFD” to “after washout” using a Sankey plot (Figure 4B). Viral representative contigs found in more than half of the individuals before the diet (n = 66) demonstrated stable presence: all of them were identified in multiple individuals in the two subsequent time points. The number of contigs shared by more than half of the individuals increased upon transition from the first (n = 66; 0.2%) to the second (n = 115; 0.3%) to the third (n = 182; 0.5%) time point. Similarly, the number of less abundant but non-unique contigs (shared by >1 individual and present in <50% of samples) increased from 4,800 (11.9%) before the diet to 5,867 (14.6%) on GFD and 5,638 (19.0%) after the washout. A similar dynamic was observed at the level of VCs (Figure S7D). Expansion of the number of shared contigs after the washout is further supported by the decrease in between-individual Bray-Curtis distances after washout (Figure S7E). Despite the individual specificity of the viral communities, post-diet between-individual distances were smaller than pre-diet between-individual distances (Wilcoxon test, p value = 0.0009; Figure S7E). This suggests that a common dietary pattern can increase the similarity of the virome composition between individuals.

**Figure 5:**
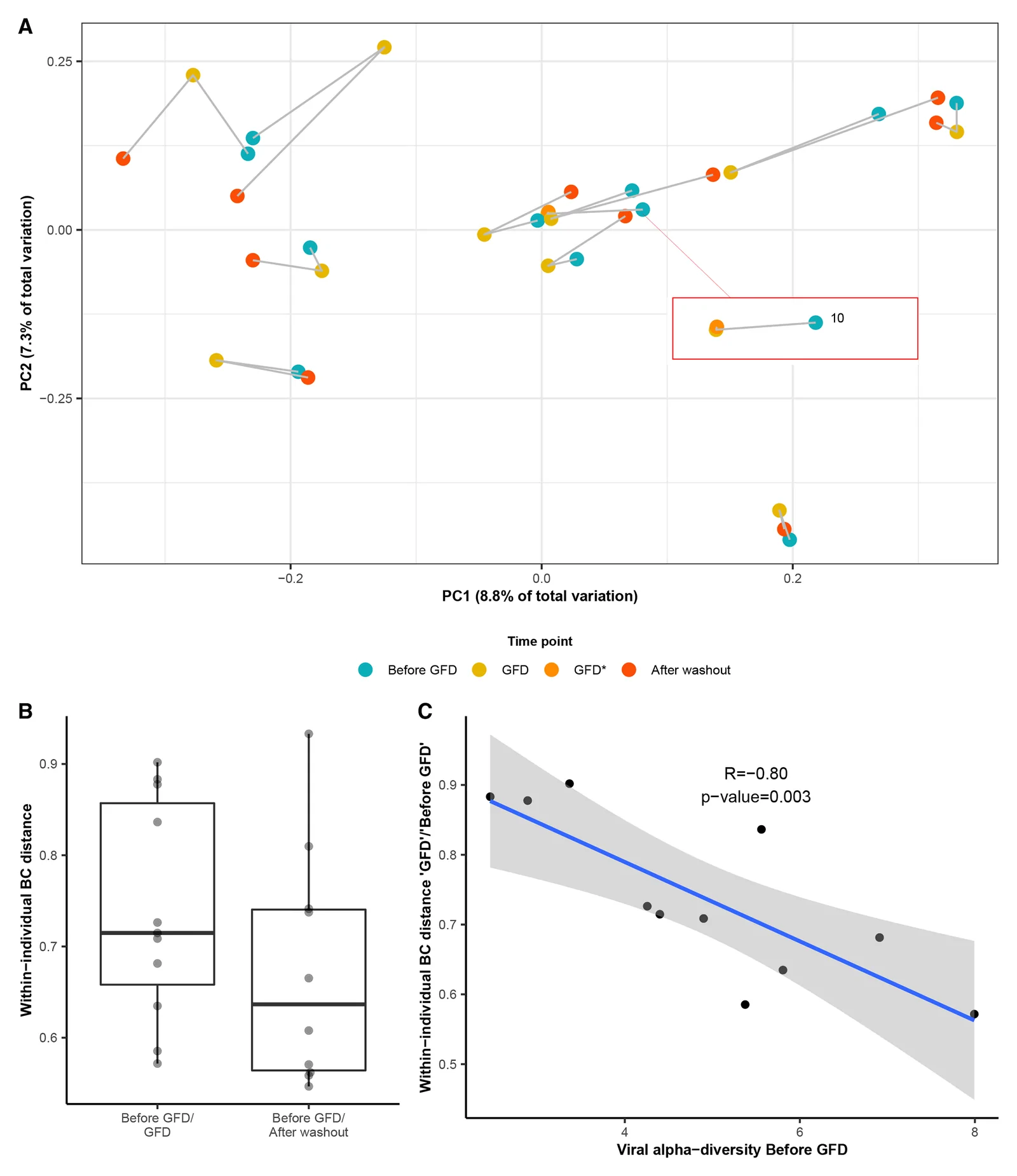
Changes in beta diversity of the human gut virome during a GFD at the level of representative contigs (A) Principal components analysis (PCA) of Bray-Curtis distances within individual time points for virome at the level of representative contigs. Gray lines connect samples from the same individuals. Individual no. 10 (inset outlined in red) was sampled twice during the GFD, and the second GFD time point is shown in dark orange. (B) Bray-Curtis within-individual distances between the time points “GFD” and “before GFD” and between “after washout” and “before GFD.” (C) Correlation between the viral alpha diversity in samples “before GFD” and Bray-Curtis distances between “GFD” and “before GFD” time points (R*pearson* = −0.8, p = 0.003).

We further explored the dynamics of beta diversity changes in the fecal virome during the GFD intervention (Figure 5A). We observed that the virome composition in GFD samples showed a large shift away from the initial composition (Figure 5A), and then became more similar to baseline after the washout period (Figure 5A). Even though Bray-Curtis distances between the time points “GFD” and “before GFD” and the time points “after washout” and “GFD” did not differ significantly (Figure 5B; p = 0.15, Wilcoxon paired test), the observed trend suggests that the human gut virome partially recovered from a GFD effect after the washout period. Subject 10 was sampled twice on the GFD (with a 2-week interval), and both samples taken during the GFD showed the lowest dissimilarity in terms of virome composition (Figure 5A, expanded inset). Overall, intra-individual (within individuals) Bray-Curtis distances for the virome were much smaller than inter-individual (between individuals) distances (Figures 5B and S7E, p < 2.2 × 10^−16^), which again confirms the high individual specificity of the viral communities.

We further investigated the role of the initial virome composition in the effect of the dietary intervention on the gut virome. Consistent with the notion of individual-specific viromes, we did not observe a consistent effect of the GFD on the virome alpha diversity (Figure 3B). However, the initial viral alpha diversity was negatively correlated with the Bray-Curtis distance between the time points “before GFD” and “GFD” (r = –0.8, p = 0.003) and explained a substantial proportion (64%) of the variance of Bray-Curtis distance between these 2 time points (Figure 5C). This indicates that the viromes of individuals with a lower initial alpha diversity were more affected by the GFD intervention, which is consistent with findings from other environmental ecosystems, suggesting that species diversity could be one of the factors that determines ecosystem resilience and responses to environmental changes (Ives and Carpenter, 2007).

To confirm that the observed changes are related to the GFD, we compared our results to the results from the longitudinal study of fecal viromes of 10 individuals over 1 year (Shkoporov et al., 2019). In the absence of any dietary intervention, no correlation was observed for the Bray-Curtis distance between 2 time points 1 month apart and the viral alpha diversity at the first time point (r = 0.003, p = 1.0, matched for seasonality).

In summary, we observed a trend toward the effect of a GFD at the level of the viral-representative contigs, and this effect was connected to the diversity of viral communities before a GFD.

### Combining custom viral databases facilitated identification of viruses

**Figure 6:**
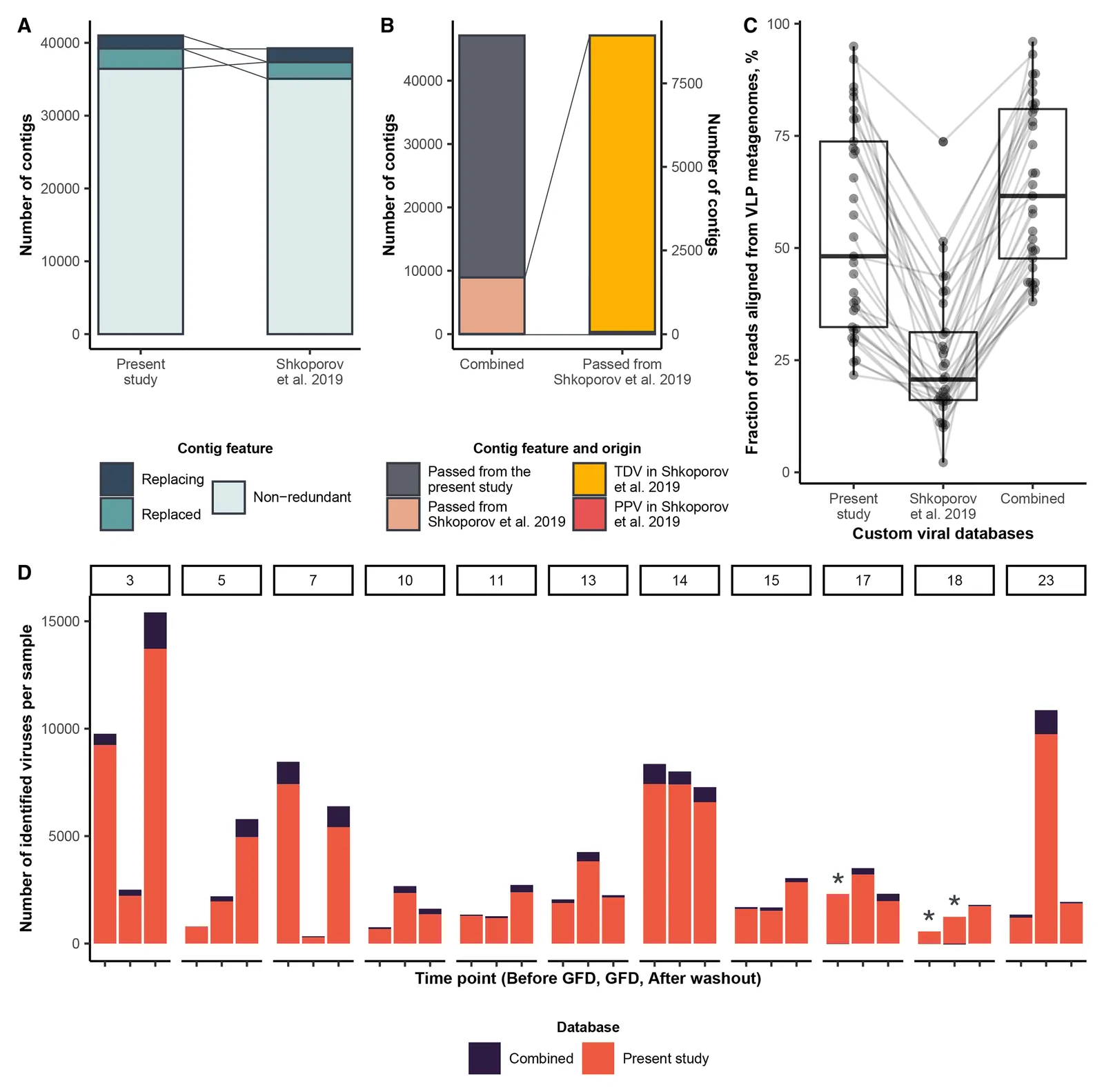
Combination of custom viral databases facilitates the virus identification (A) Proportion of contigs from the present study and the Irish study that have 90% nucleotide identity over 90% of the length of the shorter contig. The contig categories “replacing” and “replaced” are assigned based on our redundancy removal procedure (see method details). “Replacing” means we preserved the (longer) contig for the downstream analysis. “Replaced” means we removed the contig. (B) Structure of the combined viral database after pooling custom viral databases and features of contigs from the Irish study that passed the detection cutoff. Note that PPV and TDV statuses of the contigs here were derived from Shkoporov et al. (2019). (C) Fraction of quality-trimmed reads per sample aligned to contigs from the used custom viral databases and the combined curated viral database. All boxplots are standard Tukey type; see STAR Methods for details. (D) Changes of richness in samples after combining custom viral databases from the present study and Shkoporov et al. (2019). Asterisks indicate samples in which the number of identified viruses decreased after the use of the combined database.

As the VLP metagenomes showed a moderate read-mapping rate to the custom viral database of reconstructed viral genomes and fragments (median of 48.2% per sample; Figure S1), we further aimed to increase the number of mapped reads from every sample to better resolve human gut virome dynamics. We thus investigated whether combining the custom viral databases from two different populations could improve the number of mapped reads from VLP metagenomes. To do so, we pooled the viral-representative contigs from the custom databases of the present study (n = 41,014) with those from the longitudinal Irish study (Shkoporov et al., 2019) (n = 39,254). Removal of overlapping contigs from the pooled set (see method details) resulted in a combined database of 75,149 unique viral-representative contigs (Figures 6A and 6B). Among the 5,119 redundant contigs that were removed, 2,805 viral-representative contigs from the present study (6.8% if the total number of all contigs, Figure 6B) were replaced by 1,893 longer contigs from the Irish study (Shkoporov et al., 2019). After combining the 2 custom databases, the number of reads mapped from VLP metagenomes from the present study increased by an average of 9.9% per sample (Figure 6C).

Of the 75,149 viral-representative contigs, 47,136 passed the detection limit (>75% of contig coverage by reads; Figure 6B). The use of this combined database resulted in an increase in the number of viruses detected by 241 (9.6%) per sample on average (p = 5.8 × 10^−9^; Figure 6D). While the viral richness increased for 30 samples, we also observed a slight decrease in richness in 3 samples (Figure 6D). The latter finding can be explained by the fact that 32.5% of the longer contigs from the Irish study that replaced shorter contigs from the present study did not pass the detection cutoff. The total number of detected viral contigs from the Irish study that did not have homologs (at ≥90% identity over 90% of length, see method details) among contigs from the present study was 7,650; 99.2% of these contigs were assigned as TDV in the Irish study, confirming the hypothesis that TDV is more shared across individuals than PPV (Figure 6B). A total of 7.6% of the PPV and 19.4% of the TDV in the Irish study were detected among novel contigs. Of the 7,650 novel contigs, 18.3% were shared across 3 time points of at least 1 individual from the present study, and 15.4% represented individual singletons.

The increase in the number of viral genomes and fragments detected in most samples did not affect the overall dynamics of the human gut virome. We observed small changes in intra-individual Bray-Curtis distances after the increase in the number of viruses per sample (Wilcoxon paired test, p = 0.05; median δ 0.07). The correlation between initial alpha diversity and the virome composition shifts in response to the GFD was also replicated (r = -0.79, p = 0.003). These findings show that combining the viral contigs discovered in different studies increases the number of identified viruses per individual.

## Discussion

In this study, we analyzed human gut virome dynamics in relation to a GFD intervention by examining the gut viral communities in 33 samples from 11 healthy volunteers before and during a 4-week GFD and after a 5-week washout period.

In general, the detection of viruses in metagenomes is challenging. The reasons for this include the absence of universal phylogenetic markers comparable to bacterial 16S rRNA, the scarcity of the existing viral reference databases, and the high divergence of viral genome sequences. Given these challenges, we used several strategies to obtain clean viral sequences and a comprehensive overview of their diversity. First, we extracted nucleic acid from VLPs separated from bacteria by physical filtering to sequence clean viral sequences. This resulted in sequencing data with low (median 6%) bacterial contamination. Second, we included the extraction and analysis of RNA viruses, which are rarely studied in metagenomic datasets given their perceived low abundance in the human gut. Third, we performed our sequencing without using the amplification step, which allowed the accurate quantification of viruses. Finally, we applied a *de novo* assembly-based approach for virus detection (Clooney et al., 2019; Shkoporov et al., 2019) that allowed us to identify a large number of viral sequences that have not yet been deposited in existing databases and to minimize contamination by cellular DNA and RNA sequences.

As a result, we reconstructed 41,014 viral genomes and genome fragments, only 225 of which had close homologs in the Viral RefSeq database. More than 90% of the contigs were 1–25 kb in length, and this predominance of short-representative contigs suggests that a considerable proportion of reconstructed genomes is incomplete, since the average size of viral genomes of the gut is expected to be ~40–50 kbp (Hatfull, 2008). It is thus important to bear in mind that this incompleteness of the majority of the viral genomes could affect the alpha diversity metrics and our analyses based on these metrics. Using a combination of tools for virome annotation, we were able to increase the number of annotated viruses to 10,666 at the family taxonomy rank. In line with the literature, we identified several dominant gut virus families that were present in all samples, including *Siphoviridae, Microviridae*, and *Myoviridae*. Overall, the approaches described above enabled us to identify a diverse and dynamic viral community with, on average, >2,000 viral genomes per individual.

By comparing the gut virome across samples collected from different individuals, we confirmed previous findings that viral communities of the human gut are highly individual specific and dominated by a PPV comprising a minor fraction of the individual viral richness (Shkoporov et al., 2019). Only 0.3% of viral-representative contigs were shared by >50% of samples at the first time point, and within-individual Bray-Curtis distances were much smaller than between-individual distances, pointing to the high individual specificity of viromes. Longitudinal study design further allowed us to characterize the persistence of viruses in individuals throughout the study. The viruses that were most prominent across PPVs were members of the families *Siphoviridae, Myoviridae*, and *Podoviridae*. This observation is in contrast to the results from a previous study (Shkoporov et al., 2019), in which *Microviridae* and crAss-like phages were the most prominent members of PPVs. Persistent viruses composed a minor fraction of all of the viruses identified per sample (13.1% per sample on average), but they did occupy the largest proportion of the sequencing reads per sample (median 63.6%). This is consistent with the results of the previous study in healthy individuals, in which only a small subset of viruses were shared among 6 of 12 time points and determined as a PPV that recruited >90% of VLP sequencing reads per sample (Shkoporov et al., 2019). More than half of all viruses detected per individual were singletons, with an average relative abundance of 12.3% per sample, raising the question of the role of these viruses in the human gut ecosystem. For example, a higher number of singletons were previously associated with ulcerative colitis in mice (Duerkop et al., 2018), although no connection to the pathogenicity of these singletons was reported. Overall, these observations confirm the individual specificity of the human gut virome and the predominance of persistent bacteriophages and their temporal stability.

We further explored changes in the virome composition in relation to a GFD. For the viral family rank, no significant findings remained after multiple testing correction. However, we observed changes in the abundance of three viral families, crAss-like, *Podoviridae*, and *Virgaviridae*, at a nominal significance of p < 0.05. As expected, the relative abundance of viruses from the family *Virgaviridae*, which is known to infect plants, including gluten-containing species such as wheat, barley, and rye, decreased on the GFD compared to the gluten-containing diet at the first time point. At the level of representative contigs, we observed a trend toward compositional changes in the human gut virome induced by a GFD, with Bray-Curtis distances between the “after washout” time point and the “before GFD” time points being smaller compared to the “GFD” time point. However, these trends require confirmation using larger datasets. Consistent with the findings of Minot et al. (2011), post-diet between-individual distances were smaller than pre-diet between-individual distances, suggesting that the dietary intervention may have shifted the viral communities to a new state. Importantly, we observed that a lower initial diversity of the viral community was associated with larger changes in the virome upon the dietary intervention. This is in line with previous observations for the bacteriome, in which high richness is considered to reflect a stable gut community that is less prone to dietary or environmental perturbation (Coyte et al., 2015; Ives and Carpenter, 2007). These findings suggest the overall resilience of the gut ecosystem toward a dietary intervention. It is necessary to note that these results have been obtained for healthy individuals without gut-related complaints. Studies of microbiome and virome dynamics, and the effect of the diet, are important for understanding the role of the gut ecosystem in individuals with celiac disease and gluten sensitivity (Pearlman and Casey, 2019). In addition, larger studies that include information on other factors that influence microbiome and virome composition are needed to draw conclusions about bacterial-viral dynamics in relation to gluten interventions.

Studying the human gut virome often requires the use of whole-genome amplification, which may introduce biases into the representation of ssDNA viruses. Therefore, sequencing VLP metagenomes without amplification gave us the unique opportunity to investigate the virome composition and estimate the relative abundances of ssDNA circular viruses from the viral families *Circoviridae, Inoviridae*, and *Microviridae*. In other longitudinal studies, *Microviridae* was predominant in the human gut, although it was suggested that this was most likely a result of amplification bias (Lim et al., 2015; Minot et al., 2013). Our results show that even though *Microviridae* is present in all our study participant’s guts, its relative abundance was lower than described previously and comparable to the relative abundance of *Siphoviridae*. Although little is known about the relative abundances of the viral families *Circoviridae* and *Inoviridae* in the human gut, several previous studies reported that *Circoviridae* abundance was altered in malnutrition and type 1 diabetes (Reyes et al., 2015; Zhao et al., 2017). Our data suggest that the abundances of *Circoviridae* and *Inoviridae* are very low in healthy individuals, but more quantitative studies are needed to disentangle their role in health and disease.

To characterize the gut RNA virome, we applied the reverse transcription reaction to the extracted VLP nucleic acid before sequencing and used RdRp-based identification of RNA virus contigs in the downstream data analysis. In line with the literature, RNA viruses made up a small fraction of identified viruses (0.4% of all taxonomically assigned contigs) and included viruses of plant, human, and unknown hosts (Liang et al., 2020a, 2020b; Wolf et al., 2018; Zhang et al., 2006). The majority of the dsRNA and ssRNA viruses we identified belonged to the families *Picobirnaviridae* and *Virgaviridae* and were present in 15 and 26 samples, respectively. These were also previously shown to be prevalent RNA viruses of the human gut (Mukhopadhya et al., 2019). Picobirnaviruses have been linked to diarrhea in humans (Ganesh et al., 2012), although their exact hosts, pro- or eukaryotic, remain elusive (Delmas et al., 2019; Krishnamurthy and Wang, 2018; Legoff et al., 2017).

One of the major challenges in human gut virome studies is the lack of a complete viral genome database. A significant fraction of the sequencing reads from our VLP metagenomes remained unmapped (an estimated median 51.8% per sample). This is in striking contrast to the percentage of unmapped reads to the databases of known viruses, which reach up to 99% (Aggarwala et al., 2017). By combining the custom viral database of reconstructed viral contigs from our study with that of an independent study of a similar size (Shkoporov et al., 2019), we were able to increase our read mapping rate by 9.9% per sample and increase the number of identified viruses per individual by 9.6%. Our study thus shows that despite the individual-specific feature of human gut viromes, the inclusion of viral contigs reconstructed from an unrelated dataset can improve sequencing read assignment and virus identification.

In conclusion, we performed an unbiased and accurate analysis of the gut virome in 33 samples without performing whole-genome amplification. We report a large, diverse, and individual-specific gut virome community that is highly divergent across individuals. We further show that the effect of a specific diet on the human gut virome depends on the initial viral diversity and composition—in other words, the dietary intervention had less influence on a more diverse virome. By combining our virome database with an independent database, we improved the identification of viruses by 9.6%, highlighting the value of international efforts to generate reference gut viromes to improve the virus assignment and obtain the most comprehensive picture of the human gut virome composition and dynamics.

## Star⋆Methods

### Key Resources Table

**Table:** 

REAGENT or RESOURCE	SOURCE	IDENTIFIER
Chemicals, peptides, and recombinant proteins		
2-Mercaptoethanol	Sigma-Aldrich	Cat#6250
Calcium chloride	Sigma-Aldrich	Cat#793639
Chloroform, contains approximately 0.75% ethanol as preservative, for molecular biology, ≥ 99%	Fisher Scientific	Cat#10727024
DNase (TURBO)	Biosciences	Cat#AM2239
Guanidine thiocyanate solution	Sigma-Aldrich	Cat#50983
Hydrochloric acid	Sigma-Aldrich	Cat#H1758
Magnesium chloride hexahydrate	Sigma-Aldrich	Cat#M9272
Magnesium sulfate heptahydrate	Sigma-Aldrich	Cat#230391
N-Lauroylsarcosine sodium salt	Sigma-Aldrich	Cat#5125
Phenol/chloroform/isoamyl alcohol, 25:24:1 mixture, pH 6.7/8.0, ≥ 99.0%	Fisher Scientific	Cat#10306413
Polyethylene glycol 8000 (PEG 8000)	Sigma-Aldrich	Cat#P2139
Proteinase K from Tritirachium album	Sigma-Aldrich	Cat#2308
RNase1	Fisher Scientific	Cat#10568930
Sodium chloride	Sigma-Aldrich	Cat#221465
Sodium citrate tribasic dehydrate (for molecular biology > 99%)	Sigma-Aldrich	Cat#C8532
Trizma base	Sigma-Aldrich	Cat#T6066
Critical commercial assays		
1S Plus Combinatorial Dual Indexing Kit (12 × 8)	Swift Biosciences	Cat#18096
Accel-NGS 1S Plus kit	Swift Biosciences	Cat#10096
Agilent High Sensitivity D1000 ScreenTape System	Agilent Technologies	Cat#5067-5584, Cat#5067-5585
AMPure XP beads	Beckman-Coulter	Cat#A63882
DNeasy Blood & Tissue kit	QIAGEN	Cat#69506
QIAamp Fast DNA Stool Mini kit	QIAGEN	Cat#51604
Qubit dsDNA HS kit	ThermoFisher Scientific	Cat#Q32854
SuperScript IV First Strand Synthesis kit	ThermoFisher Scientific	Cat#18091200
Deposited data		
Custom viral database from (Shkoporov et al., 2019)	https://figshare.com/articles/The_human_gut_virome_is_highly_diverse_stable_and_individual-specific_/9248864 (Shkoporov et al., 2019)	N/A
chaperonin database	http://www.cpndb.ca/ (Hill et al., 2004)	N/A
COG database release 2014	https://www.ncbi.nlm.nih.gov/research/cog-project/	N/A
crAss-like phage genomic sequence database	Dr. Stephen R. Stockdale, University College Cork	N/A
NCBI nt database release 235	https://www.ncbi.nlm.nih.gov/nucleotide/	N/A
NCBI RefSeq database release 98	https://www.ncbi.nlm.nih.gov/refseq/(Brister et al., 2015)	N/A
PFAM 32.0 RdRp profiles from the CL0027 clan	https://pfam.xfam.org/clan/RdRP (El-Gebali et al., 2019)	N/A
pVOGs database	Grazziotin et al., 2017	N/A
Raw sequencing data	https://ega-archive.org/ega/home	EGA: EGAS00001005225
Supplemental datasets	https://figshare.com/s/4c76930c0792793fb2e3	N/A
Software and algorithms		
alluvial V0.1-2	https://github.com/mbojan/alluvial (Bojanowski and Edwards, 2016)	N/A
APE v5.3	(Paradis and Schliep, 2019	N/A
base v3.5.2	(R Development Core Team, 2018	N/A
BBMap v38.76	https://sourceforge.net/projects/bbmap/	N/A
BEDTools v2.25.0	Quinlan and Hall, 2010	N/A
BLAST V2.7.1+	Altschul et al., 1997	N/A
Bowtie2 V2.3.4.1	Langmead and Salzberg, 2012	N/A
Demovir	https://github.com/feargalr/Demovir	N/A
dplyr 0.8.5	https://github.com/tidyverse/dplyr	N/A
ESPript v3.0	Robert and Gouet, 2014	N/A
FastQC v0.11.7	Andrews, 2010	N/A
FigTree v1.4.2	http://tree.bio.ed.ac.uk/software/figtree/	N/A
ggplot2 v3.3.0	Wickham, 2009	N/A
HMMER v3.2.1	http://hmmer.org/(Eddy, 2011)	N/A
IQ-TREE 1.6.12	Hoang et al., 2018; Nguyen et al., 2015	N/A
KneadData v0.5.1	https://github.com/biobakery/kneaddata	N/A
MaAsLin2 v0.2.3	https://github.com/biobakery/Maaslin2(Mallick et al., 2019)	N/A
MAFFT 7.455	Katoh and Standley, 2013	N/A
MetaPhlAn2 v2.7.2	Truong et al., 2015	N/A
optparse 1.6.6	https://github.com/trevorld/r-optparse	N/A
ORFfinder	https://www.ncbi.nlm.nih.gov/orffinder/	N/A
Prodigal v2.6.3	https://github.com/hyattpd/Prodigal(Hyatt et al., 2010)	N/A
Pullseq v1.0.2	https://github.com/bcthomas/pullseq	N/A
Rv3.5.2, v3.6.2	https://cran.r-project.org/	N/A
SAMTools v1.9	Li et al., 2009	N/A
SPAdes v3.11.1	Nurk et al., 2013, 2017	N/A
stats v3.5.2	R Development Core Team, 2018	N/A
vConTACT2 v0.9.15	https://bitbucket.org/MAVERICLab/vcontact2(Bin Jang et al., 2019)	N/A
vegan v2.5-6	https://github.com/vegandevs/vegan	N/A
VirSorter v1.0.5	https://github.com/simroux/VirSorter(Roux et al., 2015b)	N/A
VRCA	https://github.com/alexcritschristoph/VRCA(Crits-Christoph et al., 2016)	N/A

### Resource Availability

#### Lead contact

Further information and requests for resources should be directed to and will be fulfilled by the Lead Contact, Prof. A. Zhernakova (a.zhernakova@umcg.nl).

#### Materials availability

This study did not generate new unique reagents.

### Experimental Model and Subject Details

The study cohort consisted of a subsample of participants who followed a GFD (Bonder et al., 2016). This subsample consisted of 11 healthy volunteers, aged 16-61. They were three males and eight females of European descent, all residents of the Netherlands, and employees or students of University Medical Center Groningen at the time of sampling (Table S5). None of the study participants had an active GI tract condition during the time of sampling. One subject (#3) received a course of antibiotic treatment during the period of observations (Table S5). Faecal samples were collected weekly from all 11 subjects throughout the study, and three samples per individual (“Before GFD,” “GFD,” and “After washout”) were selected for this study. Samples were collected in participant’s homes, transported to the laboratory and frozen immediately at –80°C. This GFD study followed the sampling protocol of the LifeLines-DEEP study (Tigchelaar et al., 2015), which was approved by the ethics committee of the University Medical Centre Groningen and conforms with the Declaration of Helsinki, document no. METC UMCG LLDEEP: M12.113965. All participants signed their informed consent prior to study enrolment.

### Method Details

#### Faecal nucleic acid extraction

The virome fraction was studied using the extraction of DNA and RNA from 0.5 g faecal aliquots, as described in Shkoporov et al. (2018). Briefly, 0.5 g of faecal material was resuspended in 10 mL of SM buffer and clarified by centrifugation (4700 rpm for 10 min at 4°C, supernatant collected and centrifuged at the same settings). Further, the supernatant was filtered twice through a 0.45 μm pore polyethersulfone membrane filter. VLPs were concentrated from the filtrate with PEG precipitation overnight and purified with chloroform treatment. The resulting fraction was treated with 8 U of TURBO DNase (Ambion/Thermo Fisher Scientific) and 20 U of RNase I (Thermo Fisher Scientific) at 37°C for 1 h before inactivating enzymes at 70°C for 10 min. Subsequently, Proteinase K (40 μg) and 20 μL of 10% SDS were added to the tubes, and incubation was continued for 20 min at 56°C. Finally, VLPs were lysed by addition of 100 μL of phage lysis buffer (4.5 M guanidinium isothiocyanate, 44 mM sodium citrate pH 7.0, 0.88% sarkosyl, 0.72% 2-mercaptoethanol) and incubation at 65°C for 10 min. Lysates were then extracted twice by gentle vortexing with equal volume of phenol/chloroform/isoamyl Alcohol 25:24:1 (Thermo Fisher Scientific), followed by centrifugation at 8000 g for 5 min at room temperature. The resulting aqueous phase was subjected to the final round of purification using the DNeasy Blood & Tissue Kit (QIAGEN) with a final elution volume of 50 μl. The total microbiome fraction was studied using DNA extraction from 0.2 g faecal aliquots with the QIAamp® Fast DNA Stool Mini Kit (QIAGEN) automated on a QIAcube with a final elution volume of 100 μL according to manufacturer’s instructions.

#### Metagenomic DNA sequencing

For sequencing of the viral fraction, 12 μL of eluted faecal VLP nucleic acid sample, regardless of concentration, was taken for reverse transcription reaction using the SuperScript IV Reverse Transcriptase (RT) kit (Invitrogen/Thermo Fisher Scientific) according to the manufacturer’s random hexamer primer protocol. DNA concentration and quality were determined using the Qubit dsDNA HS kit (Thermo Fisher Scientific). Shearing of unamplified DNA/cDNA mixture (variable amounts of DNA) was performed on an S220 Focused-Ultrasonicator (Covaris) with the following settings: peak power of 18 W, the duty factor of 20%, 50 cycles per burst, the total duration of 45 s. Further, genomic library preparation was performed with the Accel NGS 1S Plus kit (Swift Biosciences) according to the manufacturer’s instructions. Library quality was determined on an Agilent High Sensitivity D1000 ScreenTape System (Agilent Technologies) by product size and concentration. Libraries were sequenced using 2 × 150 bp paired-end chemistry on an Illumina NextSeq 550 platform (Illumina, San Diego, California) in-house at the Department of Genetics, UMCG.

For sequencing the total microbiome, DNA quality checks, library preparation using the NEBNext® Ultra II DNA Library Prep Kit for Illumina® and sequencing on a HiSeq X ten platform (Illumina, San Diego, California) with 2 × 150 bp paired-end chemistry were performed at Novogene, China.

On average, 28.3 ± 5.4 million paired-end VLP reads and 29.7 ± 4.0 million paired-end total metagenome reads were generated for each sample.

#### Quality control of metagenomic reads

Quality trimming, removal of overrepresented sequences, and read mapping to the human (hg38) reference genome was performed with KneadData (v0.5.1). On average, 18.0 ± 3.7 million paired-end VLP reads and 23.1 ± 3.1 million paired-end total metagenome reads passed quality control. The quality of the raw and clean reads was visualized with FastQC (v0.11.7). Bacterial contamination of VLP metagenomes was assessed using the single copy chaperonin gene *cpn60* database, according to Shkoporov et al. (2018).

#### Taxonomic profiling of total microbiome reads

Taxonomic profiling of the total microbiome reads was performed using MetaPhlAn2.0 (see Dataset S2 at DOI: dx.doi.org/10.6084/m9.figshare.12666830), as previously described (Kurilshikov et al., 2019).

#### Metagenomic assembly of the VLP metagenomes

Whole metagenome *de novo* assembly was performed per VLP metagenome using the settings described in Roux et al. (2019). Briefly, we performed relaxed read correction with tadpole.sh and read deduplication with clumpify.sh (BBMap, version 38.76) on quality-trimmed reads. Further, reads were assembled with SPAdes (v3.11.1) single-cell mode (error correction turned off, k-mers of 21, 33, 55, 77, 99, 127; Nurk et al., 2013). On average, 267,895 contigs were assembled for each sample, recruiting a median of 95.1% of the VLP reads per sample (Figure S1, see below for details on read mapping). Per sample, 5.4% of assembled contigs were larger than 1 kbp, which comprised 660,105 contigs when pooled for the whole dataset and recruited a substantial proportion (median 88.7% per sample) of VLP sequencing reads (Figure S1). We subjected this pooled set of contigs to a redundancy removal procedure in which contigs with 90% nucleotide identity over 90% of the length of a shorter contig were considered redundant, and the shorter contig was removed. Overall, 311,859 non-redundant or representative pooled contigs larger than 1 kbp were subject to validation as viral (see below).

#### Identifiers of samples and contigs

Samples were designated by a number assigned to an individual and a number specifying the week of sampling (Table S5), separated by a period character (e.g., 23.6). Contig identifiers included a project name (GFD) and sample designation, assembly graph node, contig length in nucleotides, and contig k-mer coverage. The latter two were omitted in text and illustrations. Importantly, a contig can be detected in samples other than the one used to assemble it and specified in its identifier.

#### Construction of the custom viral database

ORFs were predicted using Prodigal v2.6.3 in metagenomic mode. A Hidden Markov Model (HMM) algorithm (hmmsearch from HMMER v3.2.1 package) was used to search for amino acid sequences of predicted protein products against an HMM database Prokaryotic Virus Orthologous Groups (pVOGs) (Grazziotin et al., 2017). Significant hits were considered at e-value threshold of 10^−5^. Ribosomal proteins were identified using a BLASTp search (e-value threshold of 10^−10^) against a subset of ribosomal protein sequences from COG database (release 2014). VirSorter v1.0.3 (Roux et al., 2015b) along with its expanded built-in database of viral sequences (‘–db 2′ parameter) in the decontamination mode was used as one of the steps for prediction of viral sequences. Representative contigs larger than 1 kbp were considered viral if they fulfilled at least one of six criteria (similar to those described by Clooney et al. [2019] and Shkoporov et al. [2019]) or were identified with an RNA-dependent RNA polymerase (RdRp) search (described below). The six criteria were: (1) they produced BLASTn alignments to viral section of NCBI RefSeq with e-value of ≤ 10^−10^, covering > 90% of contig length at > 50% identity, (2) they had at least three ORFs, producing HMM-hits to pVOG database with e-value of ≤ 10^−5^, with at least two per 10 kb of contig length, (3) they were VirSorter-positive (all 6 categories, including suggestive), (4) they were circular (Crits-Christoph et al., 2016), (5) they produced BLASTn alignments to 427 crAss-like reference genomes (Guerin et al. [2018]; data not shown) with e-value of ≤ 10^−10^, covering > 90% of contig length at > 50% identity, or (6) they were longer than 3 kbp with no hits to the nt database (alignments > 100 nucleotides with 90% identity and e-value 10^−10^).

Contigs that had ribosomal protein genes were removed from consideration, as described in Clooney et al. (2019) and Shkoporov et al. (2019). The overall scheme is depicted in Figure S1. The final curated database of reconstructed viral sequences generated based on our dataset included 41,023 non-redundant contigs ranging in size from 1 kbp to > 221 kbp with low-to-high k-mer coverage (1–23,515.8 X, Figure S2B), which recruited 48.2% reads per sample on average.

Quality-filtered reads were aligned to 41,023 viral-representative contigs (see Table S1 and Dataset S1 at https://doi.org/10.6084/m9.figshare.12666830) on a per sample basis using Bowtie2 v2.3.4.1 in ‘end-to-end’ mode. A count table was subsequently generated using SAMTools v1.9 (see Dataset S1 at https://doi.org/10.6084/m9.figshare.12666830). Sequence coverage was calculated per contig per sample using the BEDtools v2.25.0 ‘coverage’ command (see Dataset S1 at https://doi.org/10.6084/m9.figshare.12666830). To remove spurious Bowtie2 alignments, read counts that featured a breadth of contig coverage less than 1 × 75% of a contig length were set to zero (Roux et al., 2017), resulting in 41,014 viral sequences being used for the construction of the final count table. RPKM value transformation was applied to the final count table, and the resulting RPKM count table was used in the downstream analysis.

For the combination of datasets, viral-representative contigs from Shkoporov et al. (2019) (Dataset S1, DOI: dx.doi.org/10.6084/m9.figshare.9248864) were pooled with viral-representative contigs reconstructed in the present study. From Shkoporov et al. (2019), only contigs present in the raw table counts were used (n = 39,254). The pooled set of contigs from the two studies was subjected to the removal of redundant contigs, as described above, resulting in 75,149 non-redundant viral contigs (see Dataset S3 at DOI: dx.doi.org/10.6084/m9.figshare.12666830). The procedures described above were applied to the combined viral contigs database and the quality-filtered reads from the samples from the present study to obtain the final table of counts (see Dataset S3 at DOI: dx.doi.org/10.6084/m9.figshare.12666830).

#### RdRp-based detection of RNA virus contigs

All protein sequences predicted in the non-redundant set of contigs by Prodigal 2.6.3 in the metagenomic mode were compared to the PFAM 32.0 RdRp profiles (full alignments) from the CL0027 clan (El-Gebali et al., 2019) using HMMER 3.3. Each protein longer than 50 amino acids with an RdRp profile hit characterized by an e-value < 0.001 was regarded as containing an RdRp domain unless inspection of the corresponding HMMER alignment indicated that the hit did not include key catalytic RdRp motifs. Proteins with an RdRp domain were compared to viral proteins in the NCBI RefSeq 98 database using BLASTP 2.7.1+. In each case, we retained the hit with the highest query-target percent identity among hits with > 75% query coverage and e-value < 0.001, and its target was considered as the closest homolog of the query. Multiple sequence alignments (MSAs) were built using MAFFT 7.455 (Katoh and Standley, 2013). In the case of picobirnaviruses, the MSA from Delmas et al. (2019) served as a basis. A picobirnavirus phylogenetic tree was reconstructed using IQ-TREE 1.6.12 with an automatically selected rtREV+F+R5 model, and an ultrafast bootstrap with 1000 replicates was used to estimate branch support (Hoang et al., 2018; Nguyen et al., 2015). The tree was midpoint-rooted using FigTree v1.4.2. As a control, we repeated the search for the RdRp domain in the non-redundant set of contigs translated in six frames by the EMBOSS 6.5.7 “transeq” command (Rice et al., 2000) rather than in their predicted proteins. This led to the detection of two additional contigs, GFD_15.6_NODE_1247 and GFD_18.4_NODE_366, which might represent RNA viruses divergent from recognized groups. The RdRp domains of both contigs possess canonical A and C motifs but deviate in the B motif (GxxxTxxxA).

#### Viral contig clustering

Viral contigs identified in this project, 249 crAss-like phage contigs identified in Guerin et al. (2018), and genomes of the reference database “ProkaryoticViralRefSeq97-Merged” provided with the vConTACT2 software were clustered together using vConTACT2 0.9.15 with default parameters (Bin Jang et al., 2019). Contigs assigned the status ‘Overlap’, ‘Singleton’, and ‘Outlier’ by vConTACT2 were treated as VCs consisting of a single contig in all subsequent analyses. A VC-level read counts table was generated by per-sample summation of RPKM counts for contigs belonging to each VC.

#### Taxonomy assignment of viral contigs

Family-level taxonomic annotations were assigned to viral contigs using the Demovir script (https://github.com/feargalr/Demovir) with default parameters and database. This script performs a search for amino acid sequence homologies between proteins encoded by a contigs query and a viral subset of the TrEMBL database, then uses a voting approach incorporated in the software to decide on taxonomic assignment. Demovir annotations were manually curated as follows: (a) contigs assigned as viral due to homology to the reference crAss-like genomes were pooled to the “crAss-like” family and the *Caudovirales* order despite the assignment of Demovir, (b) contigs assigned as viral due to homology to reference genomes in the Viral RefSeq database (release #98) were manually assigned to the respective families of the reference according to the coverage and identity, (c) for the contigs identified as viral based on the presence of RdRp, taxonomic assignment was made based on the nature of the closest homolog of the contig’s RdRp identified in the RefSeq database (see above), as well as compatibility of the contig length and ORF organization with the assignment, (d) Demovir assignment of the contigs GFD_5.6_NODE_3250 and GFD_3.1_NODE_293 to the RNA virus family *Flaviviridae*, as well as Demovir assignment of the contig GFD_3.6_NODE_399 to the RNA virus order *Nidovirales*, were changed to “Unassigned” based on examination of the ORF organization and protein content of these contigs, (e) Demovir assignments of contigs to the families *Ascoviridae, Baculoviridae, Iridoviridae, Marseilleviridae, Mimiviridae, Phycodnaviridae, Pithoviridae*, and *Poxviridae* were changed to “Unassigned,” as detection of these families is likely to be a result of mis-assignment (Shkoporov et al., 2019; Sutton et al., 2020), and (f) contigs from the curated viral database not reported by Demovir were marked as “Unassigned.” Next, a vConTACT2-based approach was applied to extend taxonomic assignments to more contigs. Each VC was considered. If all contigs in a VC were either assigned by Demovir to a single family (order) or unassigned to the family (order) rank, we extrapolated the Demovir assignment to the whole cluster. Otherwise, we preserved existing assignments (Tables S1 and S2).

### Quantification and Statistical Analysis

The R package *vegan* 2.5-6 was used to calculate alpha-diversity (Shannon index) and beta-diversity (Bray-Curtis dissimilarity matrices). Although the majority of the viral-representative contigs identified were 1-25 kb in length, which suggests that they may be incomplete, we did not exclude them from the calculation of alpha-diversity metrics. R package *stats* 3.5.2 was used to perform PCoA and fit linear models. The differential abundance of viral families was studied using R package *MaAsLin2* 0.2.3 with the general linear model, no transformation, and CLR-normalization as the parameters. Gender, age, and time point were chosen as fixed effects and individual as a random effect.

Illustrations were prepared using custom R scripts that employed the packages *base* 3.5.2, dplyr 0.8.5, *ggplot2* 3.3.0, *alluvial* 0.1-2 (Sankey diagram), and *APE* 5.3 (phylogenetic tree). MSAs were visualized by ESPript 3.0. All the boxplots represent standard Tukey type with interquartile range (IQR, box), median (bar), and Q1 – 1.5 × IQR/Q3 + 1.5 × IQR (whiskers).

## Supplementary Material

Supplemental information can be found online at https://doi.org/10.1016/j.celrep.2021.109132.

Supplemental information

Table S1

Table S3

Table S4

Table S5

## Data Availability

The original raw sequencing data are available from the European Genome-Phenome Archive (EGA, https://ega-archive.org/ega/home). The accession number for the original raw sequencing data is EGA: EGAS00001005225. https://ega-archive.org/ega/home The datasets generated during this study including raw count tables, contig sequences and relative abundance tables for virome and bacterial sequencing data are available at the Figshare repository under https://doi.org/10.6084/m9.figshare. 12666830. The code generated during this study is available from the Github repository (https://github.com/GRONINGEN-MICROBIOME-CENTRE/Groningen-Microbiome/tree/master/Projects/GFD_virome).

## Highlights

- Viral communities of the human gut are highly divergent across individuals
- Lower initial viral diversity is associated with greater virome response to diet
- Combining virome datasets increases the number of identified viruses per sample

## In brief

Garmaeva et al. explore the influence of a gluten-free diet on the gut virome and microbiome. They observe high variability of gut viral communities across individuals and identify a strong effect of the diet on the virome composition in individuals with lower initial viral diversity.
