# Supplementary material for: Stability of the human gut virome and effect of gluten-free diet
Source: Cell Rep. Author manuscript; Available in PMC 2026 Oct 22. (PMC7619615; doi:10.1016/j.celrep.2021.109132)
Supplement: Supplementary file 1 [file EMS217221-supplement-Supplemental_information.pdf]

Cell Reports, Volume *35*

Supplemental information

Stability of the human gut virome and effect of gluten-free diet

Sanzhima Garmaeva, Anastasia Gulyaeva, Trishla Sinha, Andrey N. Shkoporov, Adam G. Clooney, Stephen R. Stockdale, Johanne E. Spreckels, Thomas D.S. Sutton, Lorraine A. Draper, Bas E. Dutilh, Cisca Wijmenga, Alexander Kurilshikov, Jingyuan Fu, Colin Hill, and Alexandra Zhernakova

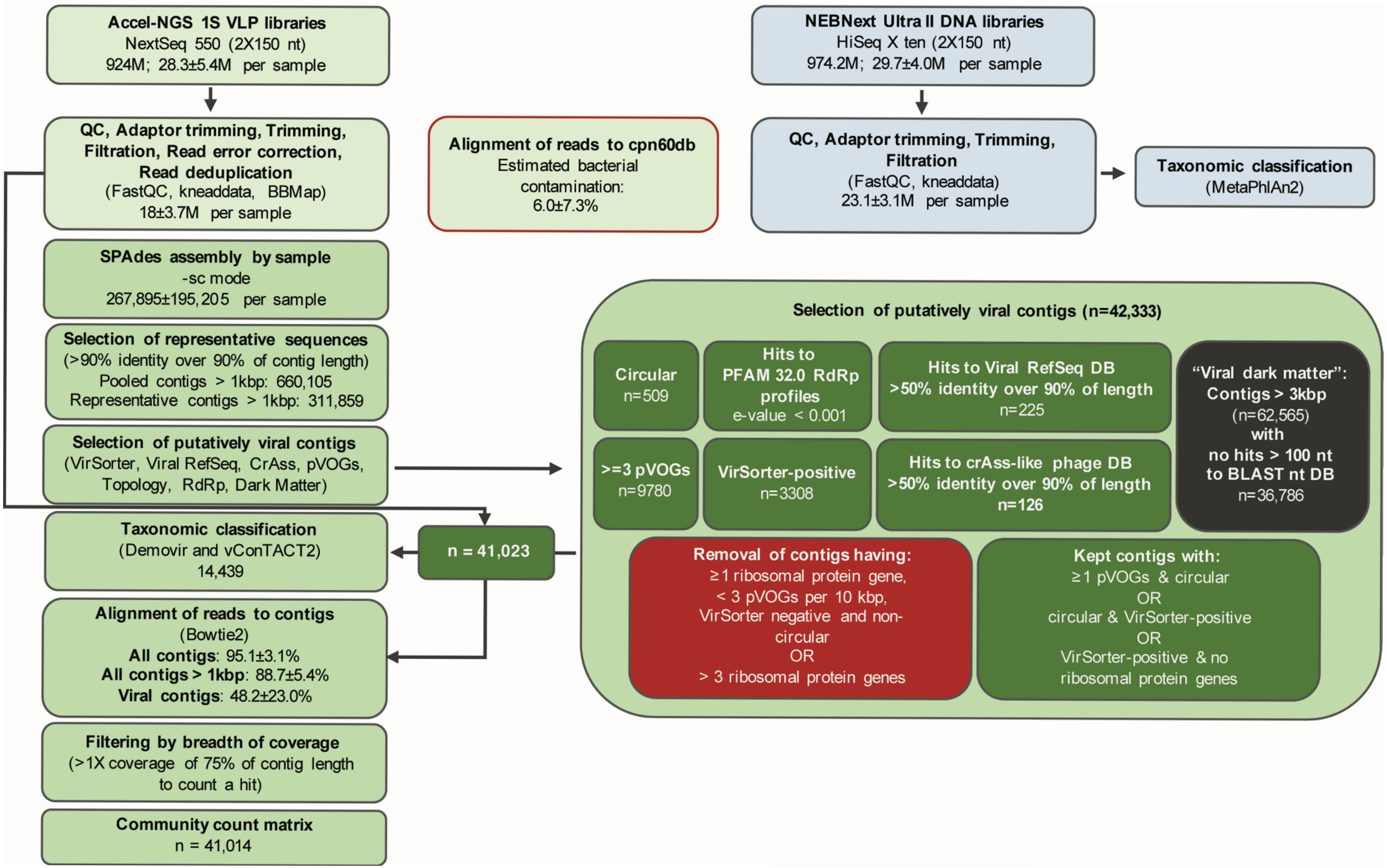

Accel-NGS 1S VLP libraries
NextSeq 550 (2X150 nt)
924M; 28.3±5.4M per sample
QC, Adaptor trimming, Trimming, Filtration, Read error correction, Read deduplication
(FastQC, kneaddata, BBMap)
18±3.7M per sample
SPAdes assembly by sample
-sc mode
267,895±195,205 per sample
Selection of representative sequences
(>90% identity over 90% of contig length)
Pooled contigs > 1kbp: 660,105
Representative contigs > 1kbp: 311,859
Selection of putatively viral contigs
(VirSorter, Viral RefSeq, CrAss, pVOGs, Topology, RdRp, Dark Matter)
Taxonomic classification
(Demovir and vConTACT2)
14,439
n = 41,023
Alignment of reads to contigs
(Bowtie2)
All contigs: 95.1±3.1%
All contigs > 1kbp: 88.7±5.4%
Viral contigs: 48.2±23.0%
Filtering by breadth of coverage
(>1X coverage of 75% of contig length to count a hit)
Community count matrix
n = 41,014
Alignment of reads to cpn60db
Estimated bacterial contamination:
6.0±7.3%
NEBNext Ultra II DNA libraries
HiSeq X ten (2X150 nt)
974.2M; 29.7±4.0M per sample
QC, Adaptor trimming, Trimming, Filtration
(FastQC, kneaddata)
23.1±3.1M per sample
Taxonomic classification
(MetaPhlAn2)
Selection of putatively viral contigs (n=42,333)
Circular
n=509
Hits to PFAM 32.0 RdRp profiles
e-value < 0.001
Hits to Viral RefSeq DB
>50% identity over 90% of length
n=225
"Viral dark matter":
Contigs > 3kbp
(n=62,565)
with
no hits > 100 nt to BLAST nt DB
n=36,786
>=3 pVOGs
n=9780
VirSorter-positive
n=3308
Hits to crAss-like phage DB
>50% identity over 90% of length
n=126
Removal of contigs having:
≥1 ribosomal protein gene,
< 3 pVOGs per 10 kbp,
VirSorter negative and non-circular
OR
> 3 ribosomal protein genes
Kept contigs with:
≥1 pVOGs & circular
OR
circular & VirSorter-positive
OR
VirSorter-positive & no ribosomal protein genes

Figure S1. Bioinformatics pipeline used for analysis of shotgun sequencing data. Related to Figure 1B. Rectangles with a green background are related to VLP shotgun sequencing data processing. Rectangles with a blue background are related to the processing of the total microbial community shotgun sequencing data. Abbreviations: nt - nucleotides; M - million; QC - quality control; kbp - kilobase pairs; DB - database. Numbers obtained per sample (i.e. number of raw and clean reads, assembled contigs, overall alignment rate, estimated bacterial contamination) are mentioned using a median with standard deviation calculated across the samples. Overall alignment rates to all assembled contigs ("All contigs"), all assembled contigs longer than 1 kbp ("All contigs >1 kbp"), putatively viral contigs ("Viral contigs") are shown in the respective rectangle.

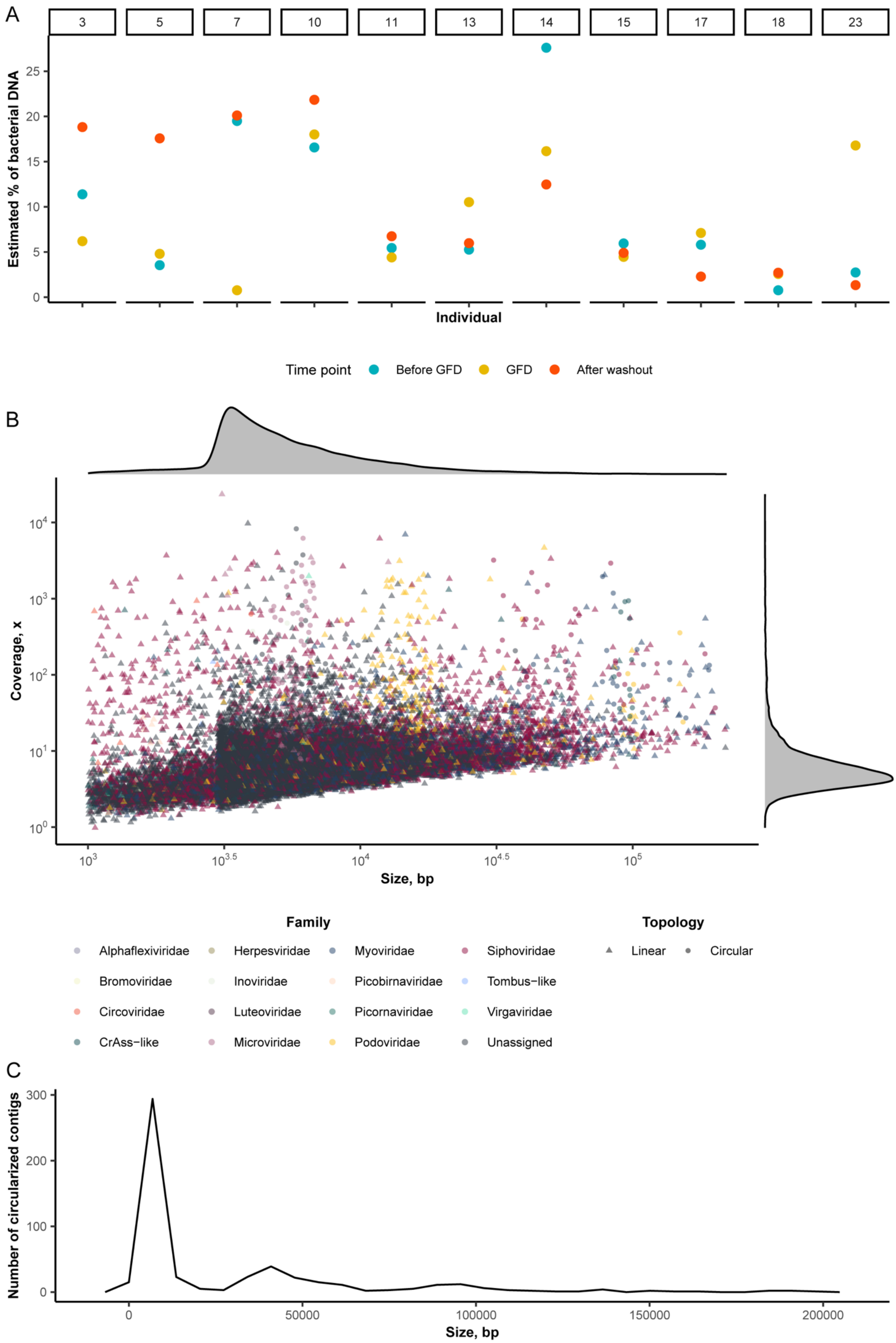

A
3
5
7
10
11
13
14
15
17
18
23
Estimated % of bacterial DNA
Individual
Time point
Before GFD
GFD
After washout
B
Coverage, x
Size, bp
Family
Alphaflexiviridae
Herpesviridae
Myoviridae
Siphoviridae
Bromoviridae
Inoviridae
Picobirnaviridae
Tombus−like
Circoviridae
Luteoviridae
Picornaviridae
Virgaviridae
CrAss−like
Microviridae
Podoviridae
Unassigned
Topology
Linear
Circular
C
Number of circularized contigs
Size, bp

Figure S2. Estimated bacterial contamination and distribution of viral-representative contigs by length and coverage. Related to Figure 1C and STAR Methods.
(A) Percentage of bacterial chromosomal DNA estimated based on the fraction of reads aligning to the conserved single-copy bacterial chaperonin gene *cpn60*. **(B)** Distribution of contigs from the curated custom database by length and depth of coverage. **(C)** Size distribution of circularized bacteriophage genomes.

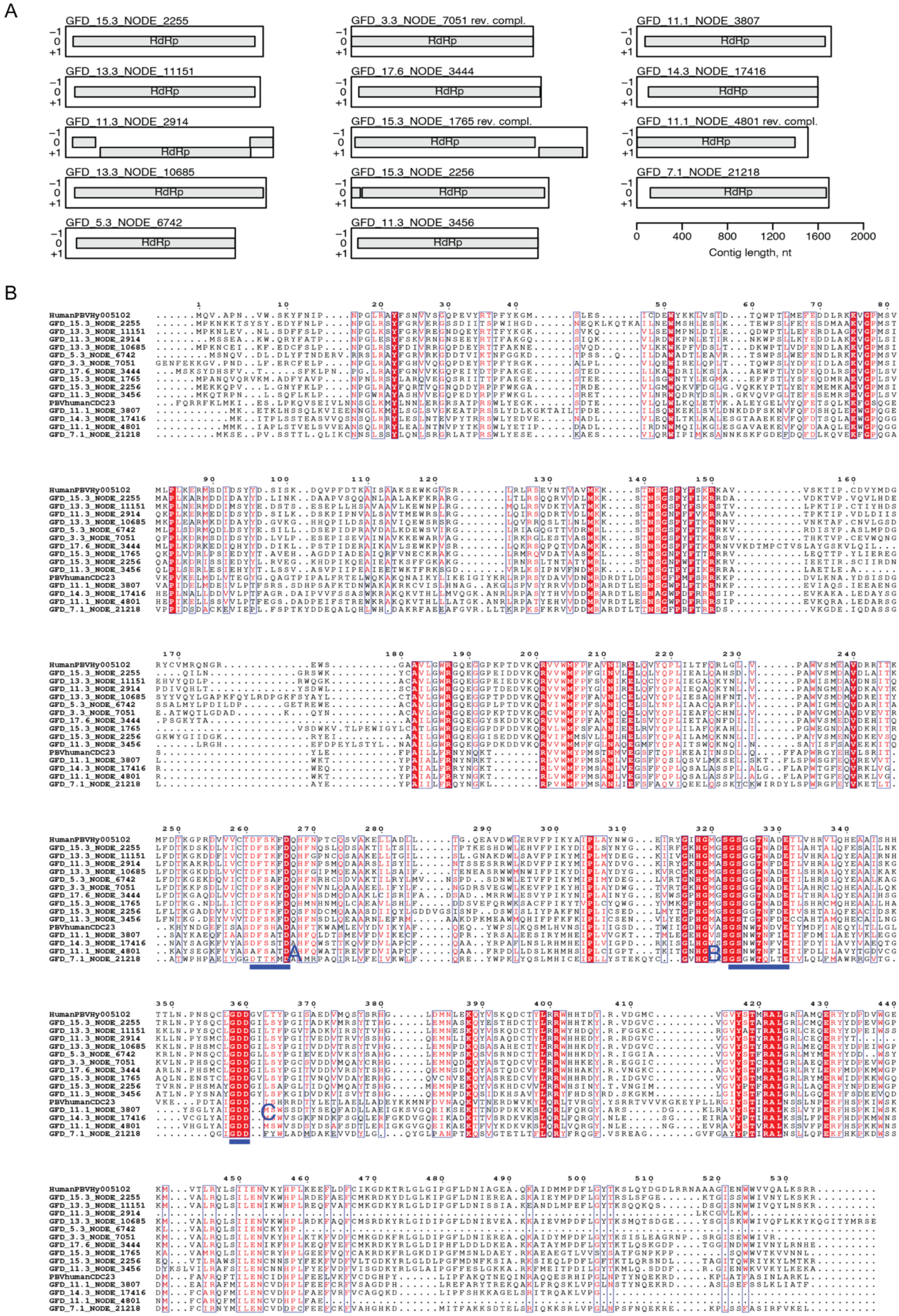
A
GFD_15.3_NODE_2255
GFD_13.3_NODE_11151
GFD_11.3_NODE_2914
GFD_13.3_NODE_10685
GFD_5.3_NODE_6742
GFD_3.3_NODE_7051 rev. compl.
GFD_17.6_NODE_3444
GFD_15.3_NODE_1765 rev. compl.
GFD_15.3_NODE_2256
GFD_11.3_NODE_3456
GFD_11.1_NODE_3807
GFD_14.3_NODE_17416
GFD_11.1_NODE_4801 rev. compl.
GFD_7.1_NODE_21218
RdRp
0
400
800
1200
1600
2000
Contig length, nt
B
HumanPBVHy005102
PBVhumanCDC23

Figure S3. ORF organization and RdRp MSA of picobirnavirus contigs. Related to Figure 2A and STAR Methods.
(A) Contigs are presented as white rectangles and ORFs predicted by Prodigal as grey rectangles. The frame is indicated relative to the 5'-terminal ORF frame, which is set to zero. **(B)** RdRp sequences of *Picobirnavidae* genogroups 1 and 2 are represented by human picobirnavirus Hy005102 (HumanPBVHy005102) and human picobirnavirus CDC23 (PBVhumanCDC23), respectively. Fourteen other sequences correspond to the contigs of this study. Absolute and partial column conservation is indicated by a red background and red font, respectively, and a blue frame. RdRp motifs A, B, and C are underlined in blue. Note that, in the protein encoded by the GFD_11.1_NODE_4801 contig, the N-terminal Asp of the motif A is substituted by Ala, which may be a sequencing artefact or a potential indicator of catalytic inactivity.

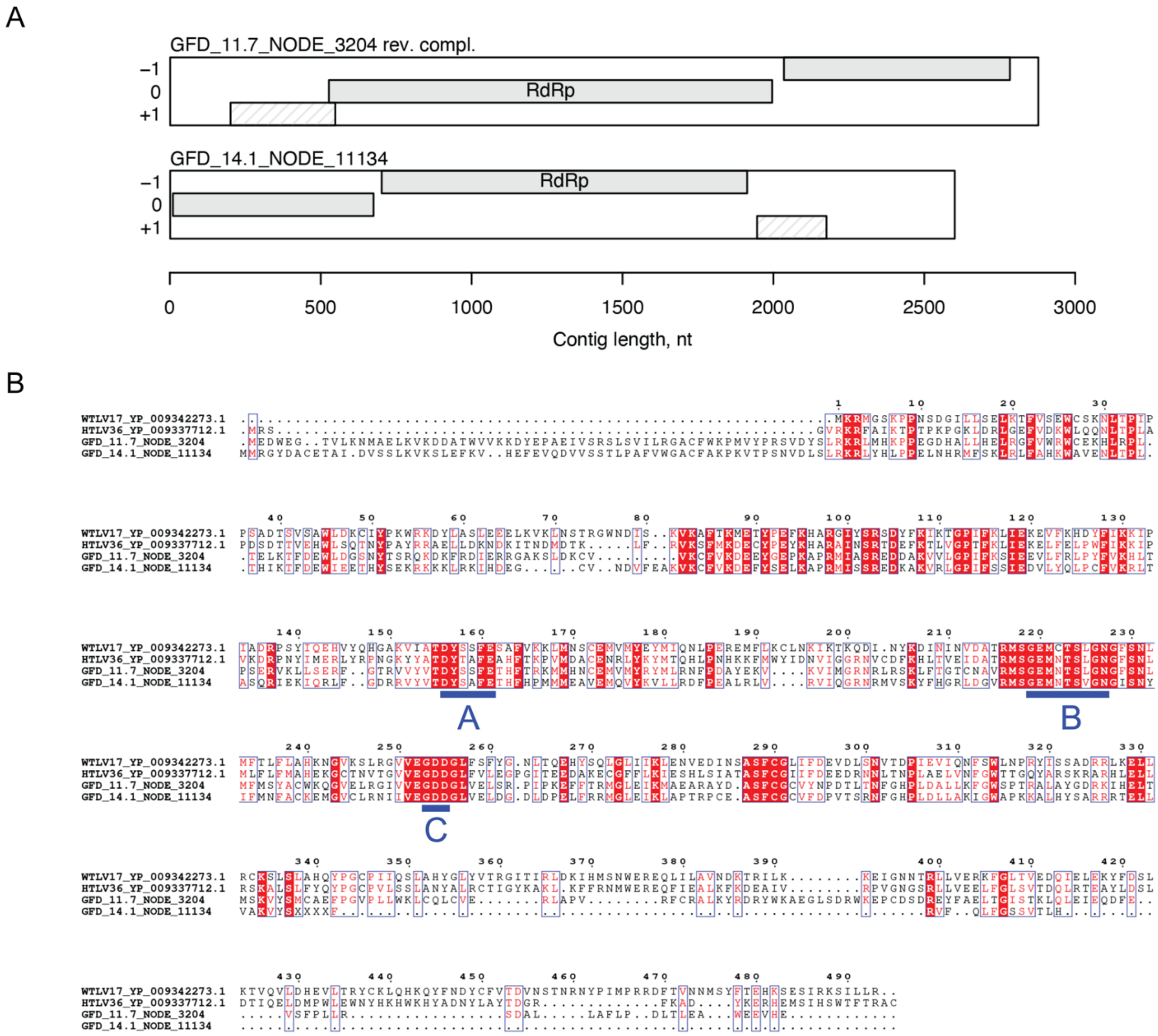

Figure S4. ORF organization and RdRp MSA of tombus-like contigs. Related to Figure 2B and STAR Methods.

(**A**) Contigs are presented as white rectangles and ORFs predicted by Prodigal as grey rectangles. The frame is indicated relative to the frame of the 5'-terminal ORF, which is set to zero. Additional ORFs >150 nt and encoded in the same strand as RdRp ORF, which were predicted by NCBI ORFfinder with default parameters, are shown by striped rectangles. **(B)** RdRp sequences of Wenzhou tombus-like virus 17 (WTLV17), Hubei tombus-like virus 36 (HTLV36), and two contigs from this study. Absolute and partial column conservation is indicated by a red background and red font, respectively, and a blue frame. RdRp motifs A, B, and C are underlined in blue. Note that all four sequences contain a variation of the canonical RdRp motif A, where Glu substitutes the C-terminal Asp.

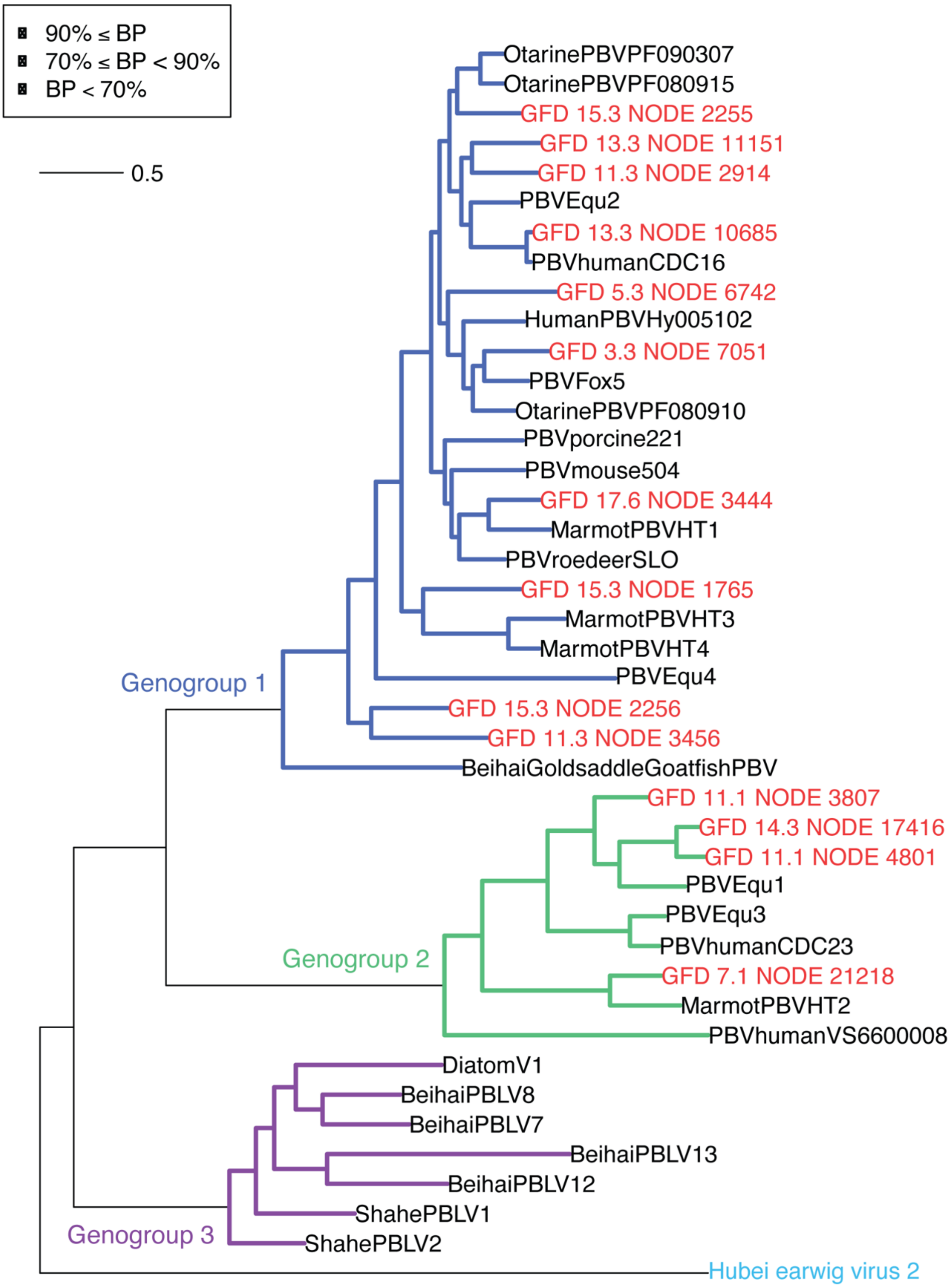

Figure S5. RdRp-based phylogeny of *Picobirnaviridae*. Related to Figures 2A and STAR Methods. The tree was reconstructed based on MSA including 27 picobirnaviruses with fully sequenced genomes from (Delmas et al., 2019). Corresponding virus identifiers are indicated in black. Identifiers of picobirnavirus contigs detected in this study are indicated in red. The name of the picobirna-like Hubei earwig virus 2 is indicated in light blue. Picobirnavirus genogroups 1, 2, and 3 are indicated by blue, green, and violet branch colours, respectively. Coloured circles indicate branch support bootstrap percentage (BP) values.

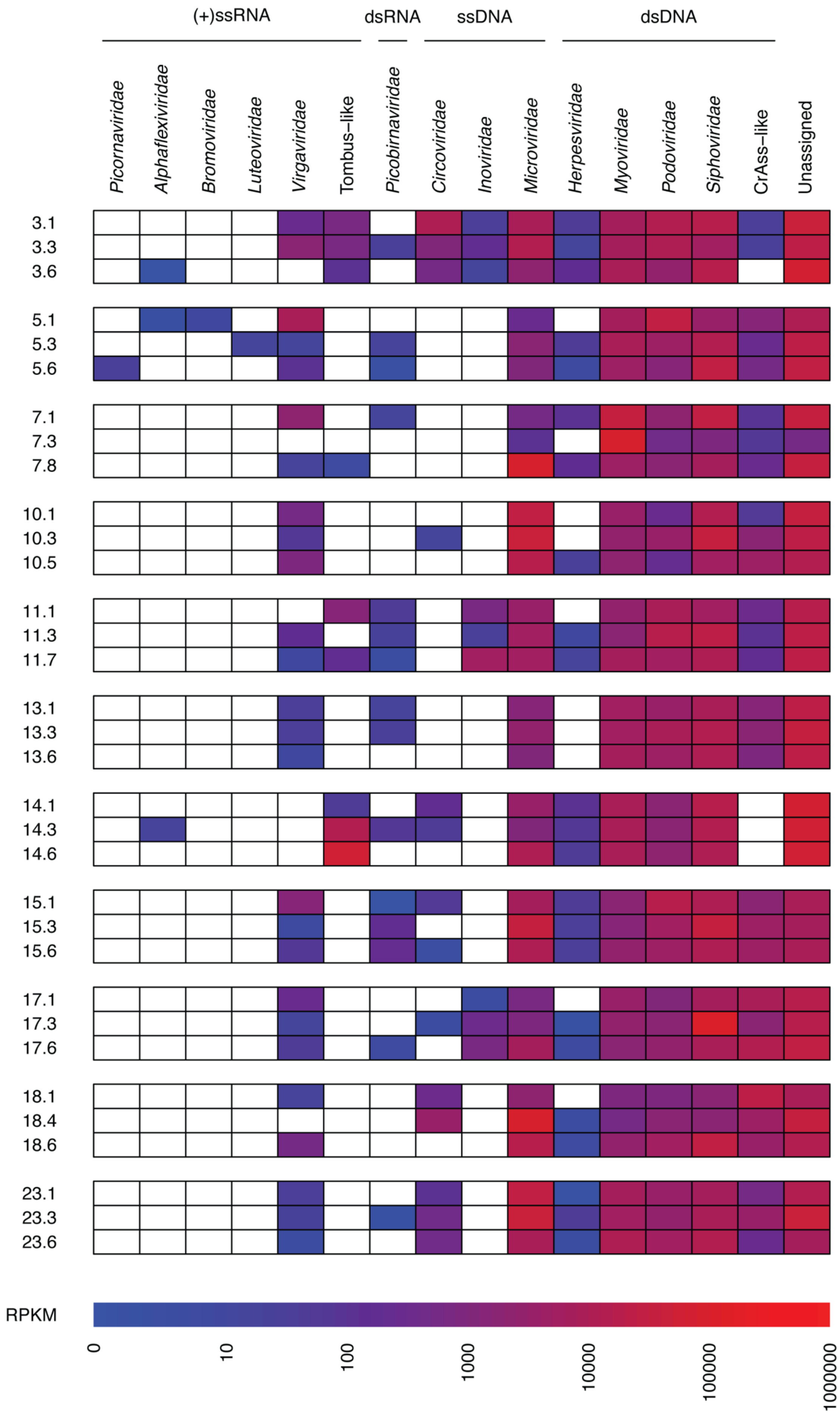
(+)ssRNA
dsRNA
ssDNA
dsDNA
Picornaviridae
Alphaflexiviridae
Bromoviridae
Luteoviridae
Virgaviridae
Tombus–like
Picobirnaviridae
Circoviridae
Inoviridae
Microviridae
Herpesviridae
Myoviridae
Podoviridae
Siphoviridae
CrAss–like
Unassigned
3.1
3.3
3.6
5.1
5.3
5.6
7.1
7.3
7.8
10.1
10.3
10.5
11.1
11.3
11.7
13.1
13.3
13.6
14.1
14.3
14.6
15.1
15.3
15.6
17.1
17.3
17.6
18.1
18.4
18.6
23.1
23.3
23.6
RPKM
0
10
100
1000
10000
100000
1000000

Figure S6. Abundance of virus families in different samples. Related to Figures 3A and 4A.
Non-zero RPKM read count values (Table S4) are indicated by colour. Families are grouped based on the virus genome organization indicated on top of the plot. Within groups, families of viruses infecting eukaryotic hosts precede those infecting prokaryotic hosts. For example, within the dsDNA group, the eukaryotic virus family *Herpesviridae* is shown before evolutionarily related bacteriophage families belonging to the order *Caudovirales* (Andrade-Martínez et al., 2019; Baker et al., 2005). Note that although 37 contigs were annotated as belonging to the order *Herpesvirales* and family *Herpesviridae*, we did not detect similarity between these contigs and herpesvirus genomes in Viral RefSeq 98 (see Methods); only two of these contigs produced protein hits with PFAM 32.0 herpesvirus profiles (HMMER 3.3, e-value <0.001).

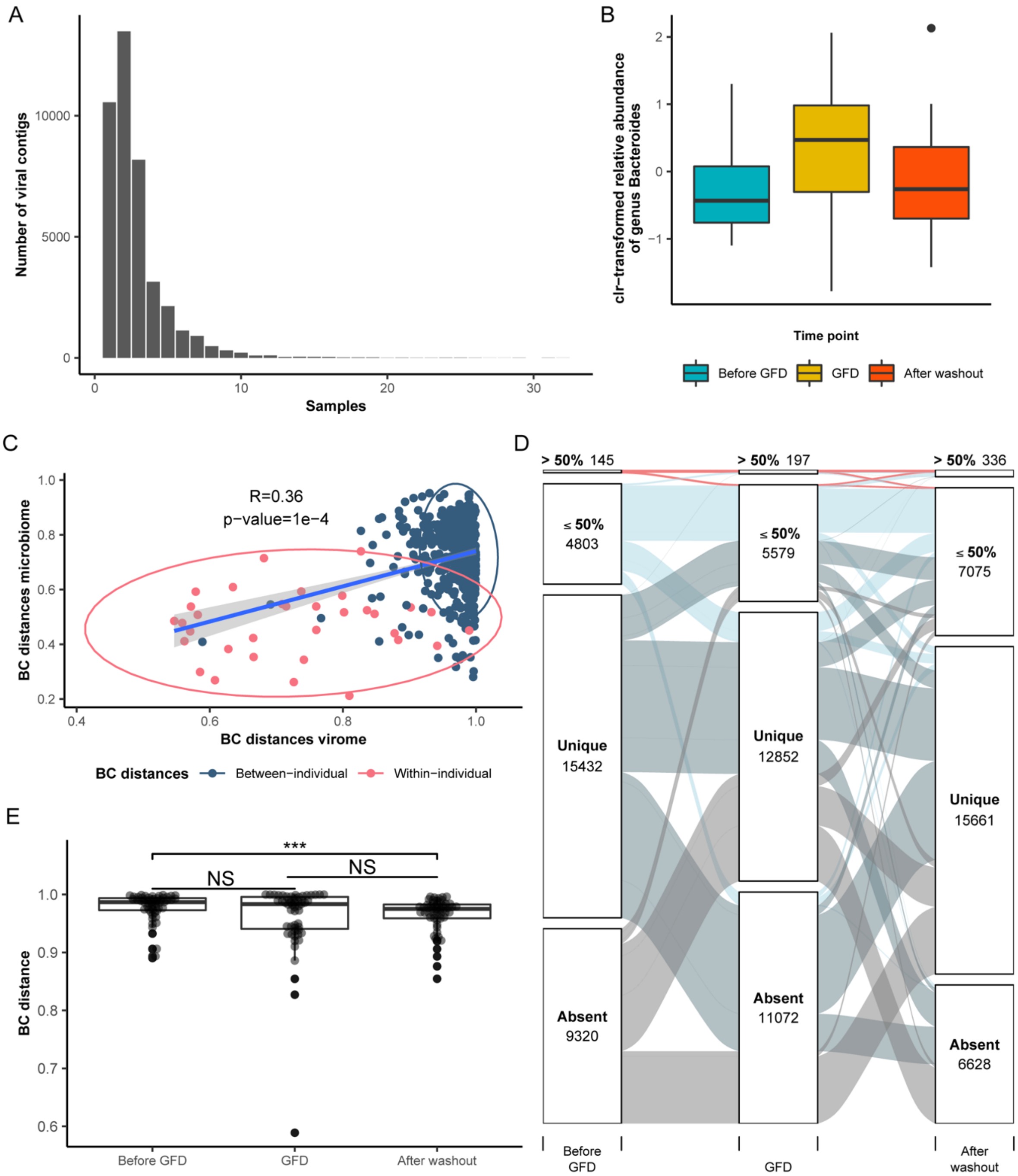

Figure S7. Human gut viral communities share a small number of viral contigs, interact with bacterial communities and have more commonality after washout. Related to Figure 4A, 4B, 5B, and STAR Methods.

(A) Number of viral contigs shared across different number of samples. **(B)** Changes of the clr-transformed relative abundance of the genus *Bacteroides*. **(C)** Covariation of microbial and viral communities β-diversity. Distance matrices for microbial and viral communities were calculated as described in Methods. The coordinates of each dot are the distances between the same samples in microbial and viral community matrices. 95% confidence intervals are shown as ellipses surrounding between-individual (dark blue) and within-individual (pink) distances. **(D)** Sankey diagram illustrates how the prevalence of VCs changed upon transition from the first to second to third time point. Category (present in >50% of samples, in ≤50% of samples, unique, absent) and number of VCs are indicated in bold and plain fonts, respectively. Samples of individual #10, who was not sampled after post-GFD washout, were excluded from consideration. **(E)** Between-individual Bray-Curtis distances at three time points.

Table S2. vConTACT2-based extension of Demovir taxonomic assignments. Related to Figures 1C, 3A, 3F, 4A, and STAR Methods.
All order and family assignments observed in the dataset are presented. Number of contigs characterized by each assignment before and after the application of the vConTACT2-based approach is specified.

**Order|Family**	**Before**	**After**
Caudovirales|crAss-like	125	133
Caudovirales|*Myoviridae*	2335	3438
Caudovirales|*Podoviridae*	522	669
Caudovirales|*Siphoviridae*	4675	6150
Caudovirales|Unassigned	3009	3772
Herpesvirales|*Herpesviridae*	12	32
Herpesvirales|Unassigned	3	3
no_order_Bromoviridae|*Bromoviridae*	2	2
no_order_Circoviridae|*Circoviridae*	9	9
no_order_Inoviridae|*Inoviridae*	4	4
no_order_Luteoviridae|*Luteoviridae*	1	1
no_order_Microviridae|*Microviridae*	161	189
no_order_Picobirnaviridae|*Picobirnaviridae*	14	14
no_order_Tombus-like|Tombus-like	2	2
no_order_Virgaviridae|*Virgaviridae*	16	16
Picornavirales|*Picornaviridae*	3	3
Tymovirales|*Alphaflexiviridae*	2	2
Unassigned|*Herpesviridae*	2	2
Unassigned|Unassigned	30117	26573
